# Recent progress in multi-walled carbon nanotube-based anode nanomaterials for high-performance rechargeable batteries

**DOI:** 10.1039/d5na01068c

**Published:** 2026-07-29

**Authors:** Yanlu lv, Md. Saiful Islam, Ghanshyam G. Tejani, Feng lin, M. A. M. Mohd Idrus

**Affiliations:** a College of Materials Science and Engineering, Guilin University of Technology Guilin 541004 China; b Department of Chemistry, Bangladesh Army University of Engineering & Technology Natore-6431 Bangladesh msaifuli2007@gmail.com; c Department of Research Analytics, Saveetha Dental College and Hospitals, Saveetha Institute of Medical and Technical Sciences, Saveetha University Chennai 600077 India; d Department of Industrial Engineering and Management, Yuan Ze University Taoyuan 320315 Taiwan; e China Nonferrous Metal (Guilin) Geology and Mining Co., Ltd Guilin 541004 China; f Universiti Kuala Lumpur, Malaysian Institute of Marine Engineering Technology (UniKL MIMET), Dataran Industri Teknologi Kejuruteraan Marin, Bandar Teknologi Maritim Jalan Pantai Remis 32200 Lumut Perak Malaysia

## Abstract

Rechargeable batteries (RBs) have become the dominant technology in the energy storage market due to their high energy density, long cycle life, and versatility in applications ranging from portable electronics to electric vehicles. However, the performance of RBs heavily depends on the anode material in which conventional graphite anodes face limitations in capacity and rate capability. Multi-walled carbon nanotube (MWCNT)-based nanomaterials have emerged as promising high-performance anodes due to their superior electrical conductivity, mechanical strength, and large surface area. Herein, recent advancements in MWCNT-based nanomaterials for RB anodes are reviewed, with emphasis on MWCNT modifications using various metals and metal oxides, their electrochemical performance, and the underlying charge-storage mechanisms. The integration of MWCNTs with various active materials such as Si, SnO_2_, Fe_3_O_4_, TiO_2_, and Co_3_O_4_ has significantly improved performance of RBs, particularly in terms of capacity, long-term stability, and charging efficiency. The metal incorporated MWCNT anode nanomaterials consistently demonstrate superior electrochemical behavior compared to traditional anode materials. This review also highlighted key strategies such as doping, hybridization with metal oxides/sulfides, and conductive polymer coatings that enhance Li-ion storage capacity, cycling stability, and performance. Challenges and future perspectives for commercializing MWCNT-based anode materials are also discussed.

## Abstract 2

Rechargeable batteries (RBs) have become the dominant technology in the energy storage market due to their high energy density, long cycle life, and versatility in applications ranging from portable electronics to electric vehicles.

## Graphical abstract

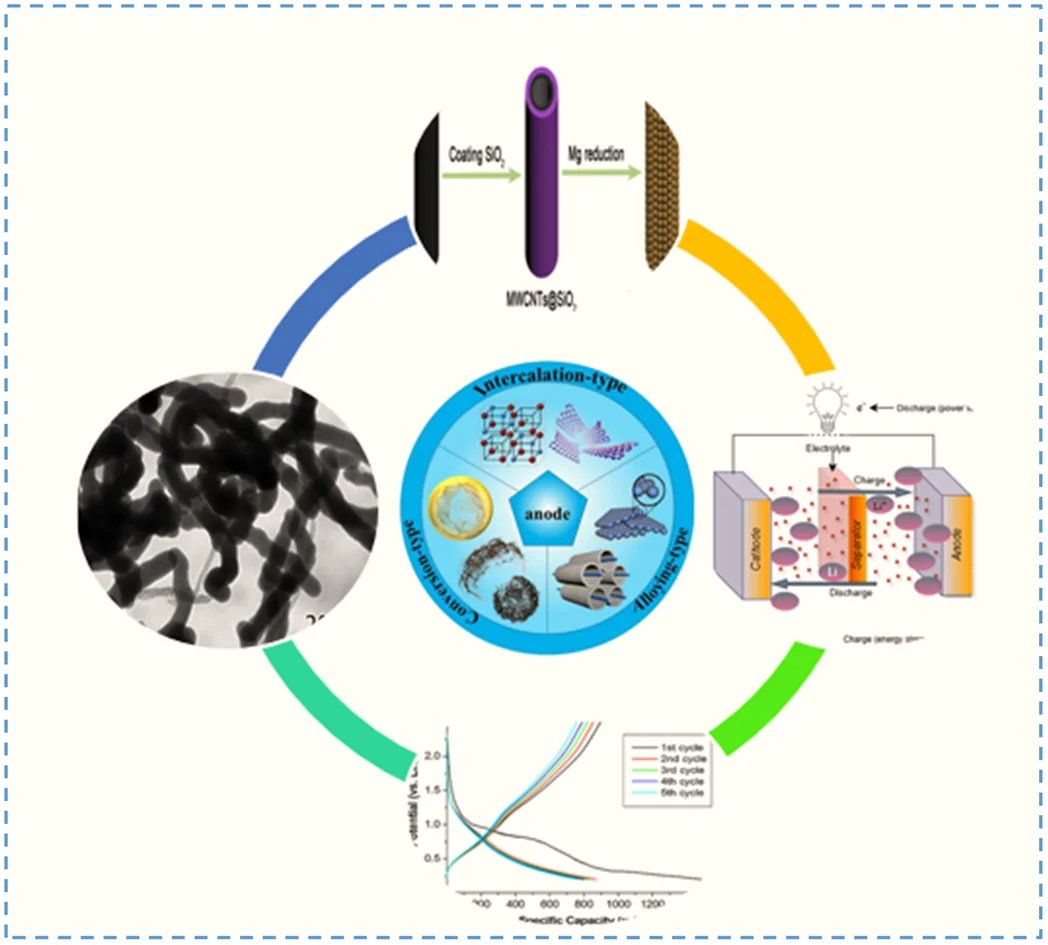

## 1. Introduction

**Fig. 1:**
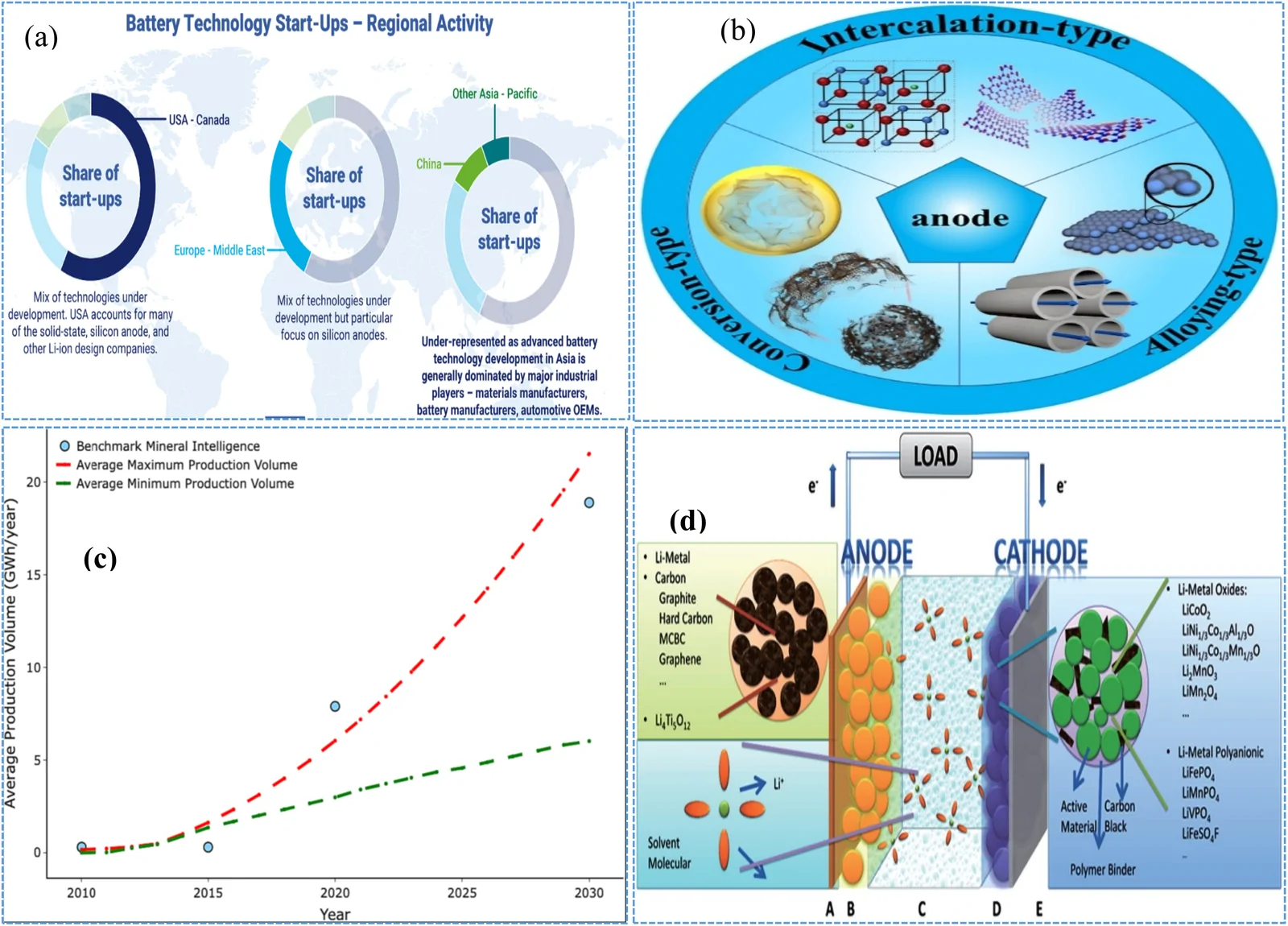
(a) Geographic distribution of battery start-up companies.^4^ (b) The three main types of anode materials for RBs.^5^ (c) A year-specific average global capacity trajectories for RB production plants based on the announced and planned manufacturer targets.^9^ (d) The construction of RBs.^10^

The rapid advancement of portable electronics, electric vehicles (EVs), and renewable energy storage systems has created an urgent demand for various high-performance rechargeable batteries *i.e.* Li-ion batteries (LIBs), nickel–metal hydride batteries, lead–acid batteries, Li–polymer batteries, sodium–sulfur batteries, sodium-ion batteries, and magnesium-ion batteries with superior energy density, long cycle life, and fast-charging capabilities.^1–5^ Since their introduction by Sony in 1990, RBs have become the dominant power source for most devices, owing to their high energy density and long cycle life.^1,2^ Other metal-ion batteries, such as zinc-ion batteries, also exhibit high energy and power densities along with long cycle life, making them highly competitive and promising candidates for advanced energy storage applications.^6^ Engineering strategies such as lattice-matched substrates, donor–acceptor COFs, and interlayer co-chemistry effectively regulate Zn deposition, redox kinetics, and ion transport in zinc-based batteries. These approaches collectively suppress dendrite growth while enhancing capacity, rate performance, and long-term cycling stability.^7,8^ However, growing global energy and mobility demands have highlighted limitations in RBs, particularly concerning energy density, safety, and longevity. The global RB market is expected to exceed US$400 billion by 2035, driven largely by the increasing demand for EVs. To support widespread EV adoption and enhance the performance of electronic devices, advancements in battery efficiency and cost reduction are essential for fueling intense competition in next-generation RB technologies. Asia, particularly China, dominates RB material processing and cell manufacturing, while the U.S. and Europe focus on building localized supply chains with strengths in innovation and advanced technologies. Start-up activity indicates strong innovation leadership in the U.S., with Europe also expanding its presence in the battery sector. Fig. 1(a) shows the regional distribution of RB technology start-ups, Fig. 1(b) presents three anode types, and Fig. 1(c) illustrates the year-wise average global RB production capacity based on announced and planned manufacturer targets. Fig. 1(d) illustrates the key components of an RB full cell, which typically consist of a current collector, anode, electrolyte, cathode, and second current collector.^3^

In recent years, RBs have attracted significant attention in both research and technology sectors due to their high energy density, long lifespan, and environmentally friendly characteristics. They are now widely recognized as the most efficient power sources for portable, flexible, and wearable electronic devices such as smartphones, laptops, and digital cameras. Compared to traditional battery technologies like alkaline, nickel–metal hydride, or nickel–cadmium batteries, RBs offer superior performance and reliability. The anode significantly influences RB performance, with graphite commonly used due to its stable layered structure that accommodates lithium ions without structural change.^11^ Performance indicators include rate capacity, coulombic efficiency, and reversible capacity, which are affected by SEI layer formation during initial cycling.^12^ Coulombic efficiency depends on lithium charge/discharge balance and can be reduced by side reactions.^13^ Enhancing power density involves minimizing electrode thickness and improving production efficiency.^14^

The performance of RBs is largely influenced by the anode material.^3,15^ In this context, silicon (Si) has emerged as a promising anode due to its exceptionally high theoretical capacity (4200 mA h g^−1^ when fully lithiated to Li_4.4_Si).^16^ Additionally, Si is the second most abundant element in the Earth's crust and environmentally benign, and benefits from mature processing technologies developed by the semiconductor industry, making it cost-effective.^17,18^ While graphite remains the dominant commercial anode material due to its stability and low cost, its limited theoretical capacity (372 mA h g^−1^) and moderate rate performance cannot meet the growing energy requirements of next-generation applications.^2^ This has driven extensive research into alternative anode materials such as silicon (Si), tin (Sn), transition metal oxides (TMOs), and sulfides (TMSs), which offer significantly higher capacities but suffer from intrinsic limitations including poor electrical conductivity and severe volume expansion during charge/discharge cycles.^3^ In this context, MWCNT-based nanocomposites have emerged as a transformative solution, combining the exceptional properties of MWCNTs with high-capacity active materials to overcome these challenges.^15^ The novelty of MWCNT-based nanocomposites lies in their unique ability to form three-dimensional conductive networks that enhance electron transport while simultaneously buffering mechanical stress from volume changes in active materials.^16^ Metal–organic frameworks (MOFs) and polyaniline (PANI) are widely used in rechargeable batteries (RBs), where MOFs enhance ion transport and surface area, while PANI improves conductivity, cycling stability, and pseudocapacitive behavior. Holey and porous heteroatom-doped graphene–silicon nanocomposites enhance lithium storage performance by preventing graphene restacking, improving ion transport, and providing strong Si–graphene interactions. These structural advantages lead to higher capacity, better rate capability, and improved cycling stability in lithium-ion batteries.^19,20^ TiNb_2_O_7_/graphene/CNT nanocomposites and CNF-enhanced MWCNT bucky-paper both demonstrate that combining carbon-based nanostructures improves conductivity, structural stability, and electrochemical performance, enabling high-rate and durable flexible energy storage systems.^21,22^ Moreover, green nanocomposites and metal halides are emerging as promising materials for advanced energy storage systems, offering sustainable or functional solutions that improve capacity, stability, efficiency, and safety in next-generation batteries.^23,24^

Recent studies have demonstrated that MWCNT-based anodes exhibit remarkable electrochemical performance, including Si/MWCNT composites achieving capacities exceeding 2000 mA h g^−1^ with 80% retention after 500 cycles,^17^ SnO_2_/MWCNT hybrids delivering ∼900 mA h g^−1^ at high current densities (mA g^−1^),^18^ and N-doped MWCNT/Co_3_O_4_ systems showing ultrahigh reversible capacity (1100 mA h g^−1^) with exceptional rate capability.^25,26^ These superior properties arise from the excellent electrical conductivity (10^3^ to 10^4^ S cm^−1^), high mechanical strength, large surface area (100–300 m^2^ g^−1^), and the hollow tubular structure of MWCNTs, which promote efficient charge transfer, Li^+^ diffusion, structural stability, and volume expansion accommodation. Their versatile surface chemistry also enables functionalization through doping or polymer coatings to further enhance performance.^27,28^ Despite these promising developments, significant challenges remain in the commercialization of MWCNT-based anode materials, including the high production cost of high-quality MWCNTs.^29^ scalability of composite synthesis methods.^30^ and long-term stability under extreme operating conditions.^31^ Recent research has focused on addressing these limitations through innovative approaches such as developing advanced binder systems to maintain electrode integrity.^32^ exploring pre-lithiation strategies to improve initial coulombic efficiency,^33^ and implementing sustainable synthesis methods to reduce environmental impact.^34^ Furthermore, the integration of MWCNT-based anodes with emerging battery technologies, including solid-state electrolytes and lithium-sulfur systems, presents exciting opportunities for future development.^35^

Despite extensive research on RB anodes, no dedicated review on MWCNT-based nanomaterials has been published to date. This comprehensive review systematically examines the latest advancements in MWCNT-based nanomaterial anodes, covering material design strategies, synthesis techniques, electrochemical performance mechanisms, and practical implementation challenges. By critically analyzing recent breakthroughs and identifying key research gaps, this work aims to provide valuable insights for the development of next-generation RB anodes that combine high energy density, excellent rate capability, and long-term cycling stability, ultimately contributing to the advancement of energy storage technologies for a sustainable future. The review also discusses future research directions and challenges for cost-effective production methods, improved understanding of interface phenomena, and the development of standardized performance evaluation protocols to accelerate the commercialization of these promising materials.

Herein, MWCNT-based anodes refer to composite electrode systems in which MWCNTs serve as the primary structural and conductive framework, governing the electrode architecture and electrochemical performance. Although additional active materials, such as silicon, transition metal oxides, or graphite, may provide the primary electrochemical energy-storage capacity, the MWCNT network plays an indispensable role in facilitating electron transport, enhancing structural stability, and accommodating volume variations during repeated charge discharge cycling.

### 1.1. The working principle of RBs

**Fig. 2:**
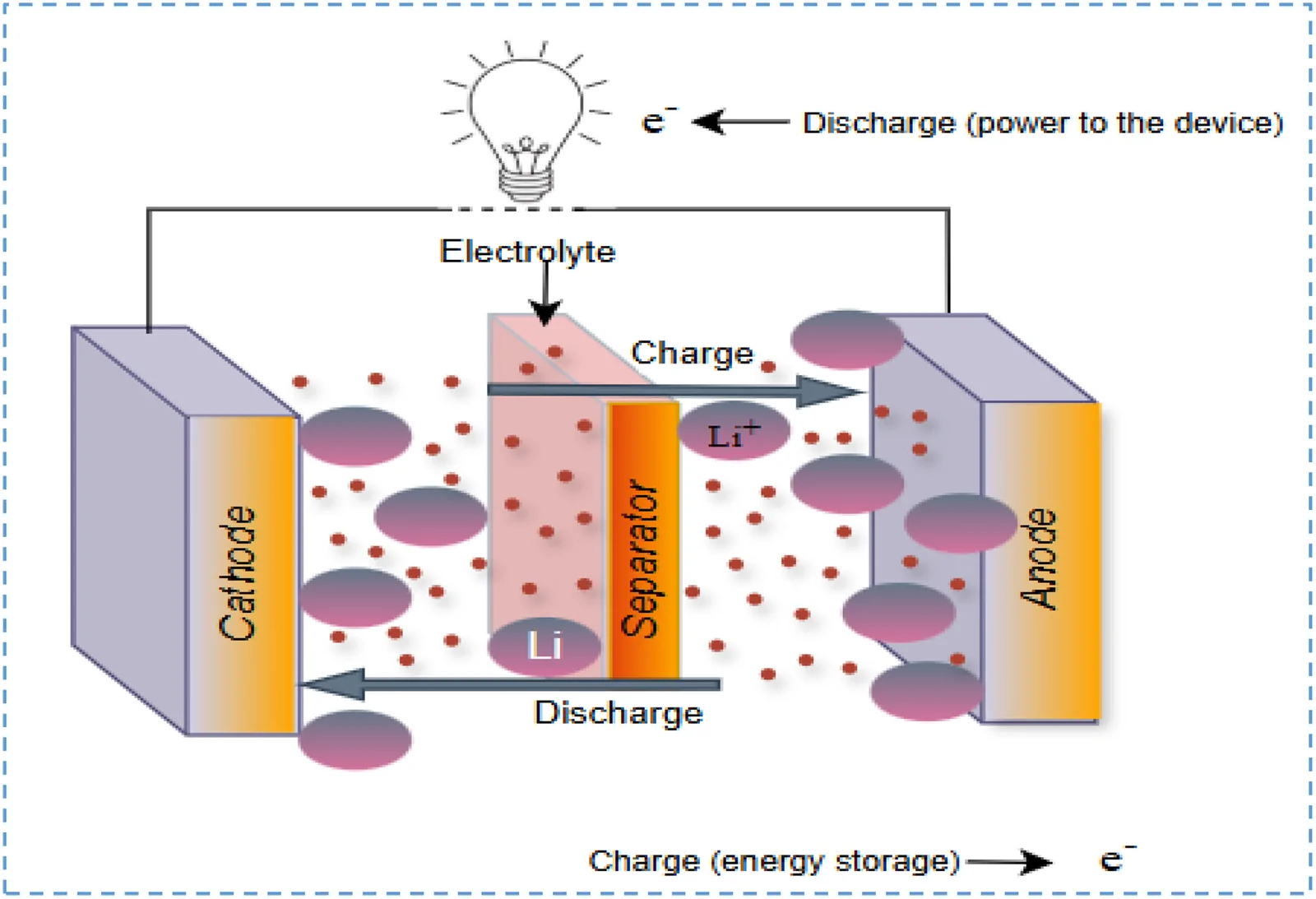
Illustrative schematic of a typical LIB as a representative RB.

RBs are energy storage devices consisting of four main components including the anode, cathode, electrolyte, and separator.^36^ Their functionality is based on the reversible movement of metal ions between the electrodes during charge and discharge cycles. As shown in Fig. 2, the separator physically isolates the anode and cathode to prevent short-circuiting while enabling Li-ion transport through the electrolyte. During the charging process, lithium ions migrate from the cathode (positive electrode) to the anode (negative electrode) *via* the electrolyte, while electrons flow through an external circuit to maintain charge balance. This process stores energy in the form of chemical potential. Conversely, during discharge, the stored lithium ions move back from the anode to the cathode, releasing the stored energy as electrical power.^37^ The efficiency of lithium-ion transport and charge transfer at the electrode interfaces significantly affects the battery's capacity, rate performance, and overall energy density. Thus, optimizing electrode materials and electrolyte systems is crucial for enhancing the performance and lifespan of RBs.^38^

Based on the working principles of RBs, the selection and development of appropriate anode materials play a crucial role in determining overall cell performance.

## 2. Anode materials

New anode materials offer the chance of significantly improved battery performance, particularly energy density and fast charge capability. Two of the most exciting material developments to Li-ions are the development and adoption of silicon anodes and Li-metal anodes, the latter often but not always in conjunction with solid-electrolytes. The excitement stems primarily from the possibility of these anode materials significantly improving energy density, where improvements of up to 50% over current state-of-the-art Li-ion cells are feasible. Enhancements to rate capability, safety, environmental profile, and even cost, are also being highlighted by silicon anode developers in particular. However, shifting from the use of silicon oxides as an additive to higher weight percentages, and the use of Li-metal anodes have posed serious problems to battery cycle life and longevity, which has delayed and limited commercial adoption so far. This report covers and analyzes the solutions being developed and provides coverage of the various companies starting to commercialize their high energy anode materials and designs. The report also provides coverage of high-rate anode materials based on metal oxides such as lithium titanate and niobium oxides. Based on the various Li-ion storage mechanisms, nanostructured anode materials for RBs are presented in Table 1.^15^

**Table 1:** Anode materials with nanostructured designs for RBs^15^

Category of anode	Sample	Reversible capacity (mA h g^−1^)/current density (mA g^−1^)/cycles	Initial discharge capacity (mA h g^−1^)/current density (mA g^−1^)
Alloy	*p*-Si@C^39^	∼1000/1000/500	1425.9/0.1
	MYS-Sn@N_*x*_C^40^	820/2000/1000	1440/0.2
	Corn-Si-1 (ref. 41)	2100/500/300	∼3500/0.2
	FP-Si@C-2 (ref. 42)	654/100/100	2364/0.1
	NiSb–C^43^	1259/100/100	1763/0.1
	NiSn–C^43^	1342/100/100	2316/0.1
	SnSe@NCNT^44^	646.2/1000/1000	1234.2/0.1
	Psi@SiO_*x*_/nano-Ag^40^	820/2000/1000	1440/0.2
	SnO_2_/CDs 300 (ref. 45)	783.3/4000/300	∼1800/0.1
Insertion	HNCNT^46^	∼250/2000/2000	>1500/0.1
	CEG–PANI (1 : 0.2)^47^	664/200/150	1531/0.2
	Li_4_Ti_5_O_12_ (LTO)^48^	150/8750/1500	181/0.175
	Nb_14_W_3_O_44_ (ref. 49)	210.1/178/200	242.6/0.178
Conversion	MnO_2_@PNC^50^	399.8/5000/100	468.4/5
	Mn doped NiCo_2_O_4_ (S-600)^51^	945/2000/500	1820/0.1
	CoO/CuO_0.43_ (ref. 52)	1668.9/100/100	1193.9/0.1
	NiO/Co_3_O_4_ (ref. 53)	668.6/1000/600	1438.1/0.1
	Mn_2_O_3_/Fe_2_O_3_ (Mn/Fe-2)^54^	750/1000/50	1403/1
	(FeCoNiCrMn)_3_O_4_HEO^55^	596.5/2000/1200	1645.3/0.1
	(Cr_0.13_Fe_0.21_Mn_0.22_Ni_0.23_Mg_0.21_)_3_O_4−*x*_ (ref. 56)	1117.6/200/300	∼600/0.1
	Nanostructured porous high-entropy oxide (NPHEO)^57^		

### 2.1. MWCNT composite anodes for RBs

MWCNTs are widely explored as anode materials for RBs, particularly in composite form, owing to their excellent interconnected conductive networks. Although pristine MWCNTs exhibit only moderate lithium storage capacity, their performance can be significantly improved through composite formation with other active materials. Moreover, MWCNTs and their composites have gained significant attention as effective anode materials for RBs due to their unique structural, electrical, and mechanical properties. MWCNTs exhibit high electrical conductivity, a large surface area, and robust mechanical strength, which collectively contribute to fast electron transport, efficient lithium-ion diffusion, and structural stability during charge–discharge cycles.^58^ However, pristine MWCNTs have limited lithium storage capacity due to their relatively inert graphitic structure. To overcome this, various composites have been developed by integrating MWCNTs with metal oxides (*e.g.*, SnO_2_, Fe_3_O_4_, and TiO_2_), silicon, or heteroatom doping (*e.g.*, N and B). These combinations enhance Li-ion adsorption, increase reversible capacity, and introduce more active sites for lithiation.^59^ MWCNTs serve as conductive frameworks that buffer volume change and prevent agglomeration of active materials, thereby improving cycle stability and rate performance.

**Fig. 3:**
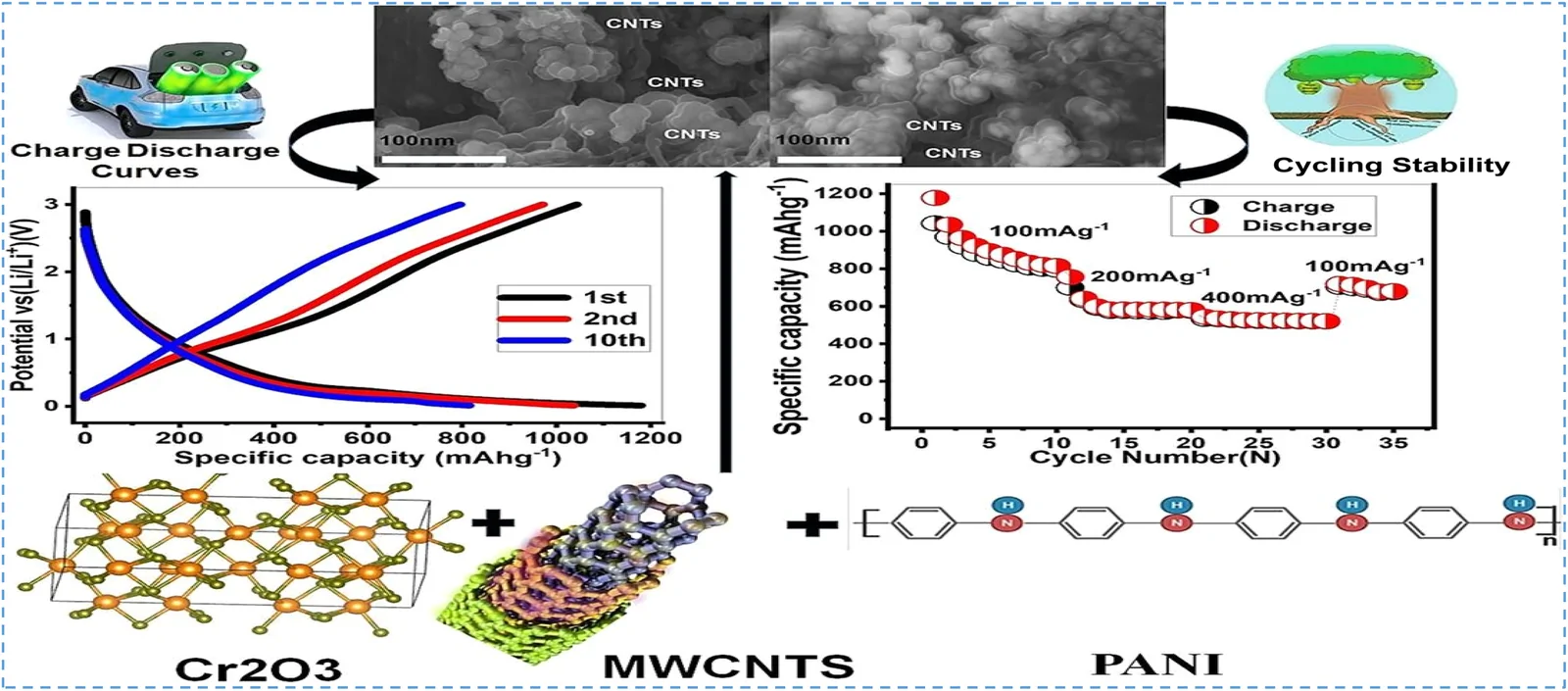
Cr_2_O_3_/MWCNTs/PANI nanomaterials as a high-performance anode material for RBs. Figure is adapted with permission from *Elsevier*.^60^

Cr_2_O_3_/MWCNTs/PANI hybrid nanomaterials as a high-performance anode material for RBs are reported by Kiran and his coauthors, and their findings are presented in Fig. 3.^60^

Fig. 3 shows that the homogeneous nanocomposite of Cr_2_O_3_-MWCNTs and PANI exhibits good electrochemical performance. The discharge capacity of Cr_2_O_3_–MWCNTs (12%)–PANI is found to be 815 mA h g^−1^ at 100 mA g^−1^, with a coulombic efficiency of 97.5%. Moreover, the nanocomposite exhibits good rate capability and cycling stability. MWCNTs consist of several concentric layers of graphene sheets, as shown in Fig. 3(a), while single-walled carbon nanotubes (SWCNTs) are made up of a single cylindrical graphene layer as shown in Fig. 3(b).^61^ MWCNTs are cylindrical shapes made up of many graphene layers that are around 0.34 nm apart from each other. They usually have sizes between 10 and 20 nm and can be several hundred microns long.^62^ Chirality vectors can be used to predict the electrical properties of single-walled carbon nanotubes (SWCNTs). However, MWCNTs have several graphene shells that may have different chiralities, which complicates their electronic behaviours. Due to their layered structure, MWCNTs allow the insertion of lithium ions in a manner akin to graphite. This property makes them particularly attractive for applications in lithium-ion batteries, as they can enhance overall capacity and cycling stability. Additionally, the unique structural characteristics of MWCNTs can lead to improved conductivity and mechanical strength, further benefiting their use in energy storage technologies. Furthermore, lithium can intercalate into both the spaces between layers and the hollow cores in the middle, which could give them a larger capacity than graphite anodes. The structural transformation of MWCNTs to graphene-like layers increases the active surface area and exposes additional lithium storage sites, thereby enhancing Li^+^ adsorption, intercalation, and charge transfer kinetics. However, excessive exfoliation may also promote SEI formation and irreversible capacity loss during cycling.

Many studies have shown that MWCNT-based anodes work very well in lithium-ion batteries.^63–66^ For example, Shin *et al.* made very pure MWCNTs under mild conditions.^63^ They had excellent reversibility and minimal hysteresis losses, but the lithium diffusion rates were only modest. The MWCNT anode, grown directly on a copper current collector *via* catalyst deposition and CVD, delivered nearly triple the capacity of graphite, stable performance at 3 (C), and no capacity loss over 50 cycles.^61^ Its enhanced properties stem from efficient Li-ion intercalation, strong substrate bonding, and high conductivity.

When lithium is added to MWCNTs, the graphene layers peel off, which increases the surface area and helps the SEI develop, which leads to a greater irreversible capacity. Zhang *et al.* reported that factors including the tube's diameter, wall thickness, and the size and concentration of solvated Li-ions affect this exfoliation.^64^ It seems that larger, thicker-walled MWCNTs are more stable since smaller-diameter tubes are more likely to break down. 3D type MWCNT architecture has also shown better performance. Kang *et al.* developed a 3D MWCNT-based anode that had a higher loading capacity and performed better than 2D anodes in terms of capacity, stability, and rate capability.^65^ Similarly, Welna *et al.* examined both vertically aligned and randomly orientated MWCNTs.^66^ They discovered that the vertically aligned MWCNTs worked better than the randomly orientated ones, mainly because they allowed electrons to move more easily and improved the flow of ions. This result indicates that the arrangement of MWCNTs plays a crucial role in optimizing the performance of energy storage devices. The aligned structures made it easier for lithium to stick to them and for electricity to flow through them. They started with a capacity of 980 mA h g^−1^ and stayed at 750 mA h g^−1^ after a few cycles. On the other hand, non-aligned MWCNTs had substantially lower capacities (158 mA h g^−1^ at first and 58 mA h g^−1^ after 10 cycles), which shows how important structural order is for electrochemical performance. Overall, lithium storage performance of MWCNT-based anodes is strongly governed by structural characteristics such as tube dimensions and alignment, where optimized architectures (*e.g.*, larger, thicker-walled, and vertically aligned MWCNTs) enhance stability, charge transport, and electrochemical capacity. A summary of various MWCNT-based anode materials reported in the literature is provided in Table 2.

**Table 2:** Various MWCNT-based anodes and their preparation methods with performances in RBs

Types of anode materials	Specific capacity (mA h g^−1^)	Cycle retention	Preparation method	Key benefits
Fe_2_O_3_/MWCNT^67–69^	654 after 200 cycles	High reversibility at 0.5 A g^−1^	Hydrothermal synthesis	Improved conductivity; volume change buffering
Fe_3_O_4_/MWCNT^69,70^	1700 initial; 850 at cycle 30	∼80% retention over 30 cycles	Hydrothermal annealing	High capacity; strain buffering
Fe_2_O_3_/COOH-MWCNT^67,68^	711.2	400 cycles at 500 mA g^−1^	Hydrothermal method	Buffers volume changes; improved conductivity *via* the CNT network
Si/MWCNT^67,71^	610 over 1200 cycles	Excellent stability at 0.5 A g^−1^	Thermal plasma synthesis	Nano-Si on MWCNTs; enhanced cycling stability
Si-Coated MWCNT tissue^67,72^	900 initial; 680 at 400 mA g^−1^	Good rate capability	Magnetron sputtering	Flexible; high coulombic efficiency
SiO_2_@MWCNT^73^	936 initial; 826 after 200 cycles	87.5% retention	Acid treatment + chemical reduction	Binder-free; enhanced dispersion and stability
PVDF–MWCNT composite^74^	255 initial; 251 after 40 cycles	∼98% retention	Vacuum filtration	Flexible, free-standing, and good mechanical strength
Sn–MWCNT^75^	589 initial; 422 at cycle 50	∼72% retention	Electrophoretic electrodeposition	3D network; superior electrochemical performance
Pure MWCNTs^67,75^	∼210–255 initial; ∼220–251 after 20–40 cycles	∼100% retention	Direct use as an anode	Stable cycling, good rate capability and compatibility with diverse cathodes
Binder-free MWCNTs on Cu^76^	∼1000	No degradation after 50 cycles	Chemical vapor deposition (CVD)	Direct growth on current collectors, high conductivity, and 3× graphite capacity
NBC@MWCNTs^77^	450 (5 A g^−1^)	90% (5000 cycles)	Hydrothermal carbonization followed by annealing	Ultra-high-rate capability
Si/MWCNT core–shell^78^	3250	99.8% (700 cycles)	CVD	Exceptional capacity and stability

MWCNT-based composites have emerged as promising anode materials for RBs, owing to their unique structural properties and enhanced electrochemical performance. Optimizing the diameter of pure MWCNTs within the range of 40–60 nm has been shown to effectively balance lithium storage capacity and structural stability. Incorporating metal oxides, such as Fe_2_O_3_, into MWCNT composites enhances overall conductivity and helps mitigate the intrinsic limitations of metal oxides, such as poor electrical conductivity and volume expansion. Additionally, binder-free composite designs remove inactive components, thereby improving energy density and simplifying fabrication. Doping MWCNTs with heteroatoms like nitrogen and boron further enhances lithium-ion storage by introducing additional active sites, while also preserving the mechanical integrity of the structure during charge–discharge cycles. This review discusses MWCNT-based nanocomposite anodes, with a particular focus on those incorporating transition metal oxides, metals, and metal sulfides.

### 2.2. Metal oxide-based MWCNT anodes for RBs

**Fig. 4:**
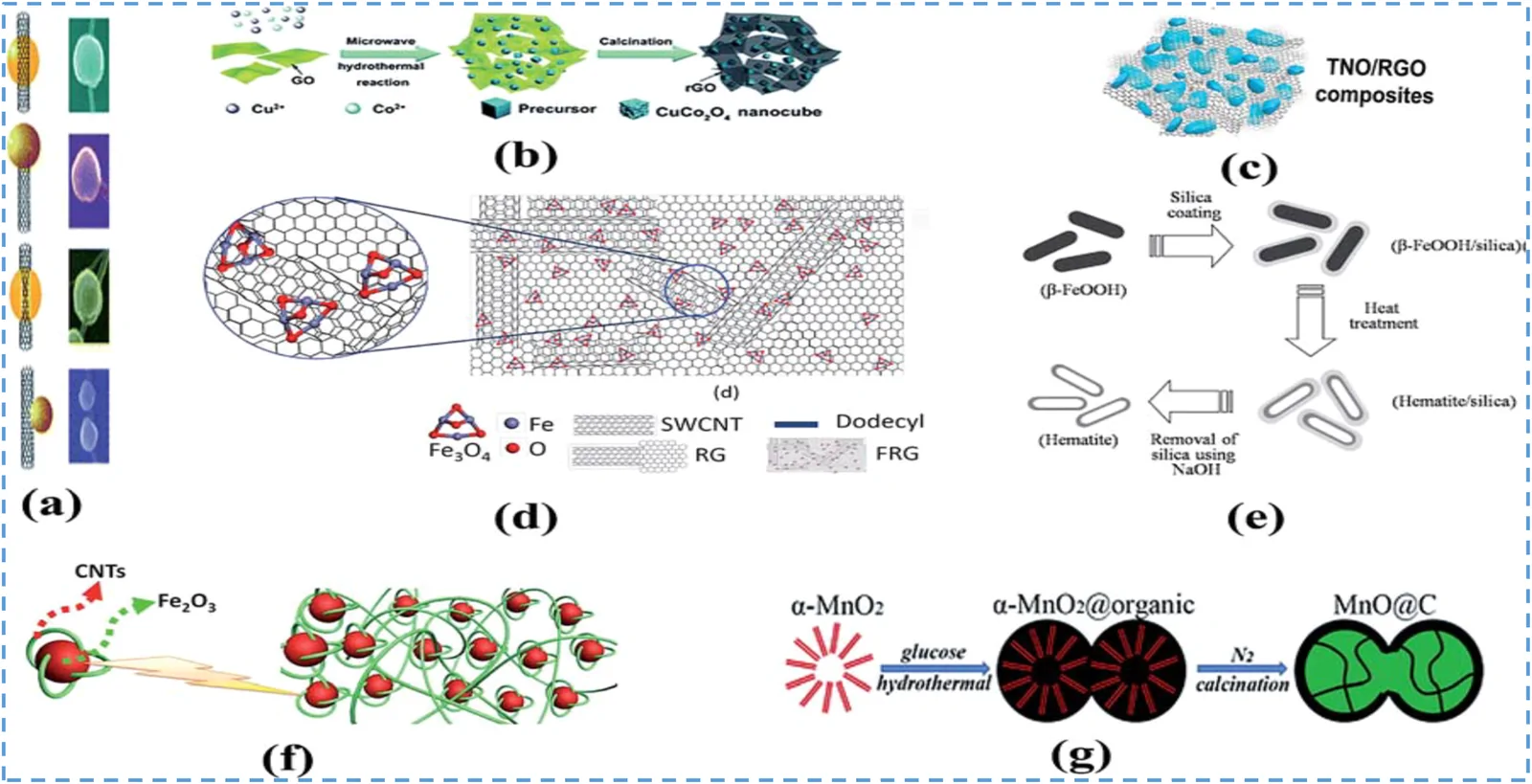
(a) Illustration of different configurations of Co_3_O_4_–CNT heterostructures. Adapted with authorization from ref. 87, © 2013, *Nanoscale*.^87^ (b) A porous CuCo_2_O_4_ nanocube/reduced graphene oxide (rGO) composite. Adapted with permission from ref. 88, © 2014, *Nanoscale*.^88^ (c) Conceptual structural diagram of a Ti_2_Nb_10_O_29_/rGO hybrid material. Reprinted with approval from ref. 89, © 2015, *J. Power Sources*.^89^ (d) Representation of a self-supporting Fe_3_O_4_/rebar graphene composite membrane. Reused with consent from ref. 80, © 2016, *J. Alloys Compd.*^80^ (e) Depiction of the method used to fabricate hematite nanocapsules using a wrapping–baking–peeling strategy. Adapted with consent from ref. 90, © 2010, *Electrochem. Commun.*^90^ (f) Illustration of hybrid architectures formed by α-Fe_2_O_3_ and CNTs. Reproduced with authorization from ref. 91, © 2015, *ACS Appl. Mater. Interfaces*.^91^ (g) Diagram outlining the synthesis of peanut-like α-MnO_2_ sphere@C core–shell composites. Adapted with permission from ref. 92, © 2014, *Nanoscale*.^92^

Metal oxide-based MWCNT nanocomposites have emerged as highly promising anode materials for RBs, offering significant improvements over traditional graphite anodes due to their superior theoretical capacities, enhanced electrical conductivity, and better structural stability.^1^ Transition metal oxides not only possess relatively high capacity and good cyclability but are also cheap and easily available. Nonetheless, the commercial application of transition metal oxide anodes is far from mature. Quite a few problems including high working potential, low coulombic efficiency and large potential hysteresis between discharge and charge are tough to handle.^79^ Previous studies demonstrated that flexible free-standing hollow Fe_3_O_4_/graphene films exhibit enhanced conductivity, structural stability, and electrochemical performance, making them promising electrodes for flexible lithium-ion batteries.^80^ This improvement is primarily due to the high electrical conductivity and buffering capability of amorphous carbon. For instance, Zhang *et al.* developed a free-standing Fe_3_O_4_/rebar graphene (FRG) composite film (Fig. 4), which showed outstanding rate capability and excellent cycling stability, benefiting from the conductive network of graphene and the elimination of binders and additives.^81^ Similarly, Zou *et al.* engineered an anode composed of Ag-doped carbon nanofibers (CNFs) embedded with Fe_2_O_3_ nanoparticles (Ag–Fe_2_O_3_/CNFs), which delivered high-rate capability and stable cycling performance, even under low-temperature conditions.^82^

The integration of TMOs such as MnO_2_, TiO_2_, Co_3_O_4_, Fe_3_O_4_, Fe_2_O_3_, Co_3_O_4_, and MoO_3_ with MWCNTs addresses the inherent drawbacks of metal oxides, including poor conductivity and severe volume expansion during charge–discharge cycles, by leveraging the high surface area, mechanical resilience, and conductive pathways provided by MWCNTs.^83,84^ Recent studies have demonstrated remarkable electrochemical performance; for instance, α-MoO_3_/MWCNT composites synthesized *via* surfactant-assisted solvothermal methods achieved reversible capacities exceeding 1000 mA h g^−1^ after hundreds of cycles, while Fe_2_O_3_/COOH-MWCNT and Co_3_O_4_/MWCNT core–shell structures have shown excellent cyclability and rate capability, with MWCNTs effectively buffering volume changes and facilitating rapid electron and ion transport.^85,86^ SnO_2_/MWCNT composites combine the high theoretical capacity of SnO_2_ (∼790 mA h g^−1^) with the excellent electrical conductivity and mechanical flexibility of MWCNTs, resulting in improved capacity retention and rate performance. Similarly, Fe_3_O_4_/MWCNTs benefit from the high capacity of Fe_3_O_4_ and the buffering effect of MWCNTs, which mitigates volume expansion during cycling, thereby enhancing cycling stability. In the case of TiO_2_/MWCNTs, although TiO_2_ inherently has a lower capacity, it offers excellent structural stability and safety. The incorporation of MWCNTs significantly improves its electrical conductivity and rate capability, making the composite suitable for stable and efficient energy storage applications. Various composites of anodes using graphene oxide (GO), CNTs and transition metal oxide are shown in Fig. 4.

Advanced synthesis strategies, including MOF-derived pyrolysis and hydrothermal methods, enable precise control over the nanostructure and composition, further optimizing lithium-ion diffusion and charge transfer. Structural innovations such as core–shell and porous architectures help minimize solid electrolyte interphase (SEI) degradation and accommodate mechanical strain, thereby enhancing cycling stability.^93^ Despite challenges like initial irreversible capacity loss and scalability concerns, ongoing research into heteroatom doping and hybrid carbon supports continues to push the boundaries of performance. Zhang *et al.* (2015) reported multi-walled carbon nanotube-reinforced porous iron oxide as a superior anode material, highlighting enhanced rate capability and long-term cycle stability due to the conductive network of MWCNTs combined with Fe_3_O_4_ nanoparticles.^94^ Li *et al.* (2023) discussed mesoporous Cr_2_O_3_/MWCNTs/PANI nanocomposites as high-performance anodes, emphasizing the role of porous structures and conductive polymer coatings in improving capacity and cycling stability.^95^ Wang *et al.* (2024) presented porous hexagonal Mn_5_O_8_ nanosheets with reduced graphene oxide composites, demonstrating excellent fast-charging capability and high reversible capacity, which is analogous to MWCNT-based composites in enhancing lithium-ion storage performance.^96^

A freestanding TiO_2_/MWCNT-anode paper was fabricated *via* vacuum filtration using a hybrid material synthesized by RF magnetron sputtering. SEM images showed a 3D nanoporous CNT network with uniformly distributed TiO_2_ nanocrystals (7.14–12.21 nm).^97^ The electrode prepared at 200 W exhibited a high specific capacity of 230 mA h g^−1^ with excellent cycle stability over 50 cycles. The MWCNTs offer flexible mechanical support and conductive pathways, while TiO_2_ contributes to high capacity. A crystalline α-MoO_3_/MWCNT nanocomposite was synthesized *via* a surfactant-assisted solvothermal method and low-temperature calcination. It shows high capacity (up to 1392.2 mA h g^−1^), excellent rate performance, and long-term cycling stability, attributed to MWCNT-enhanced conductivity and the porous structure.^98^

Nano-ZnMn_2_O_4_ and ZnMn_2_O_4_/MWCNT/graphene composites were synthesized *via* sol–gel and ball-milling methods. XRD and TEM confirmed nanosized spinel structures (∼7 nm). Electrochemical tests showed high initial capacity (∼1978 mA h g^−1^) for pure ZnMn_2_O_4_ and stable cycling (∼190 mA h g^−1^, 65% retention) for the composite over 100 cycles.^99^ Overall, metal oxide/MWCNT nanocomposites represent a transformative direction for high-capacity, durable, and scalable RB anodes, aligning with the growing demand for advanced energy storage solutions.^94–96^

SiO_2_/MWCNT nanocomposites have emerged as promising anode materials for lithium-ion batteries due to their unique structural and electrochemical properties. Coating or embedding MWCNTs with SiO_2_ improves the composite's electrochemical performance by buffering the volume changes associated with lithium insertion/extraction and preventing agglomeration. SiO_2_ contributes to a high theoretical capacity and forms a stable solid electrolyte interphase (SEI), which improves cycling stability. Moreover, the MWCNT framework facilitates fast lithium-ion diffusion and maintains electrical connectivity, compensating for the intrinsic low conductivity of SiO_2_. The synergistic combination of MWCNTs and SiO_2_ results in nanocomposites with improved specific capacity, rate performance, and long-term cycling durability, making MWCNT@SiO_2_ a strong candidate for next-generation high-performance lithium-ion battery anodes.

**Fig. 5:**
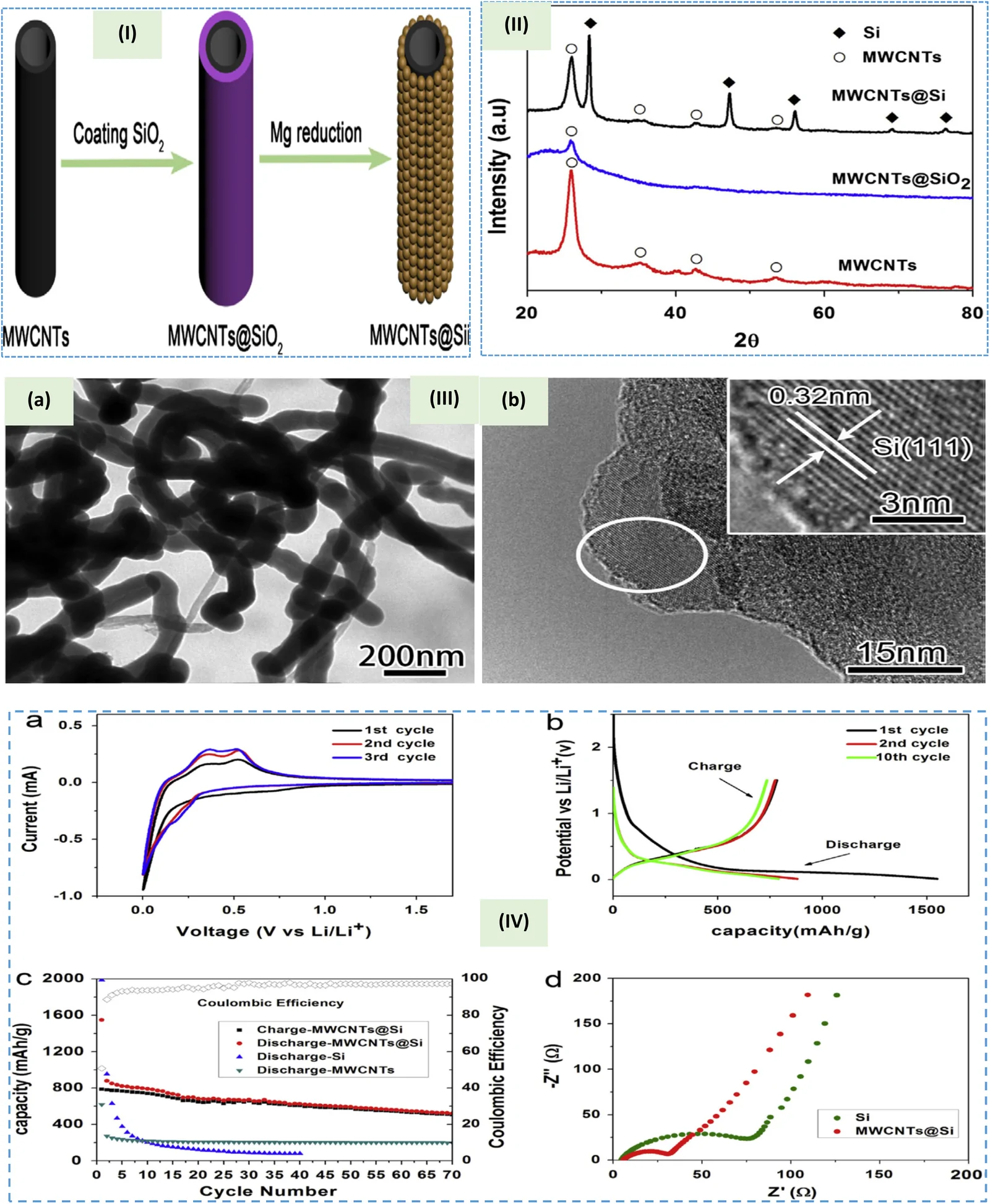
(I) Schematic of the synthesis process for uniform MWCNT@Si nanocomposites. (II) XRD patterns comparing MWCNTs, MWCNT@SiO_2_, and MWCNT@Si. (III) TEM analysis: (a) high-resolution image of MWCNT@SiO_2_ nanocables; (b) HRTEM image of MWCNT@Si. (IV) Electrochemical performance: (a) CV curves from the first three cycles; (b) voltage profiles at 400 mA g^−1^ (0.001–2 V); (c) cycling performance (capacity and efficiency) of MWCNT@Si, pure Si, and MWCNTs; (d) impedance comparison between bulk Si and MWCNT@Si. Adapted with permission from ref. 100.

Yifan Chen *et al.* conducted research on facile synthesis of uniform MWCNT@Si nanocomposites as high-performance anode materials for lithium-ion batteries.^100^ MWCNT@Si nanocomposites were effectively synthesized using the magnesiothermic reduction of pre-synthesized MWCNTs@SiO_2_ nanocables (Fig. 5). MWCNT@Si nanocomposites exhibit superior reversible capacity and cycling performance compared to bulk Si and unmodified MWCNTs (Fig. 5). The superior electrochemical performance is ascribed to the innovative porous nanostructure and the incorporation of MWCNTs, which mitigate volume fluctuations, sustain the electrical conductive network, and improve electronic conductivity and lithium-ion transport. The superior electrochemical performance is due to the innovative porous nanostructure and the incorporation of MWCNTs, which mitigate volume changes, sustain the electrical conductive network, and improve electronic conductivity and lithium-ion transport (Fig. 5(IV(a–d))). The XRD pattern shows that once the SiO_2_ layer has been coated, amorphous cristobalite can be identified by a broad peak at ∼23, whereas the peak strength of MWCNTs is weaker, confirming the presence of the SiO_2_ layer (Fig. 5(II)). After the SiO_2_ coating, the diameter of the MWCNTs increases to approximately 80–120 nm, resulting in distinct core–shell architectures, as shown in Fig. 5(III(a) and (b)). Importantly, no separate SiO_2_ nanoparticles are observed except those attached to the MWCNT surfaces, confirming the successful formation of uniform MWCNT@SiO_2_ nanocables, which subsequently lead to MWCNT@Si/SiO_*x*_@C nanocomposites exhibiting improved stability and electrochemical performance as lithium-ion battery anodes by effectively buffering silicon volume expansion and enhancing conductivity through a protective coaxial structure.^101^

**Fig. 6:**
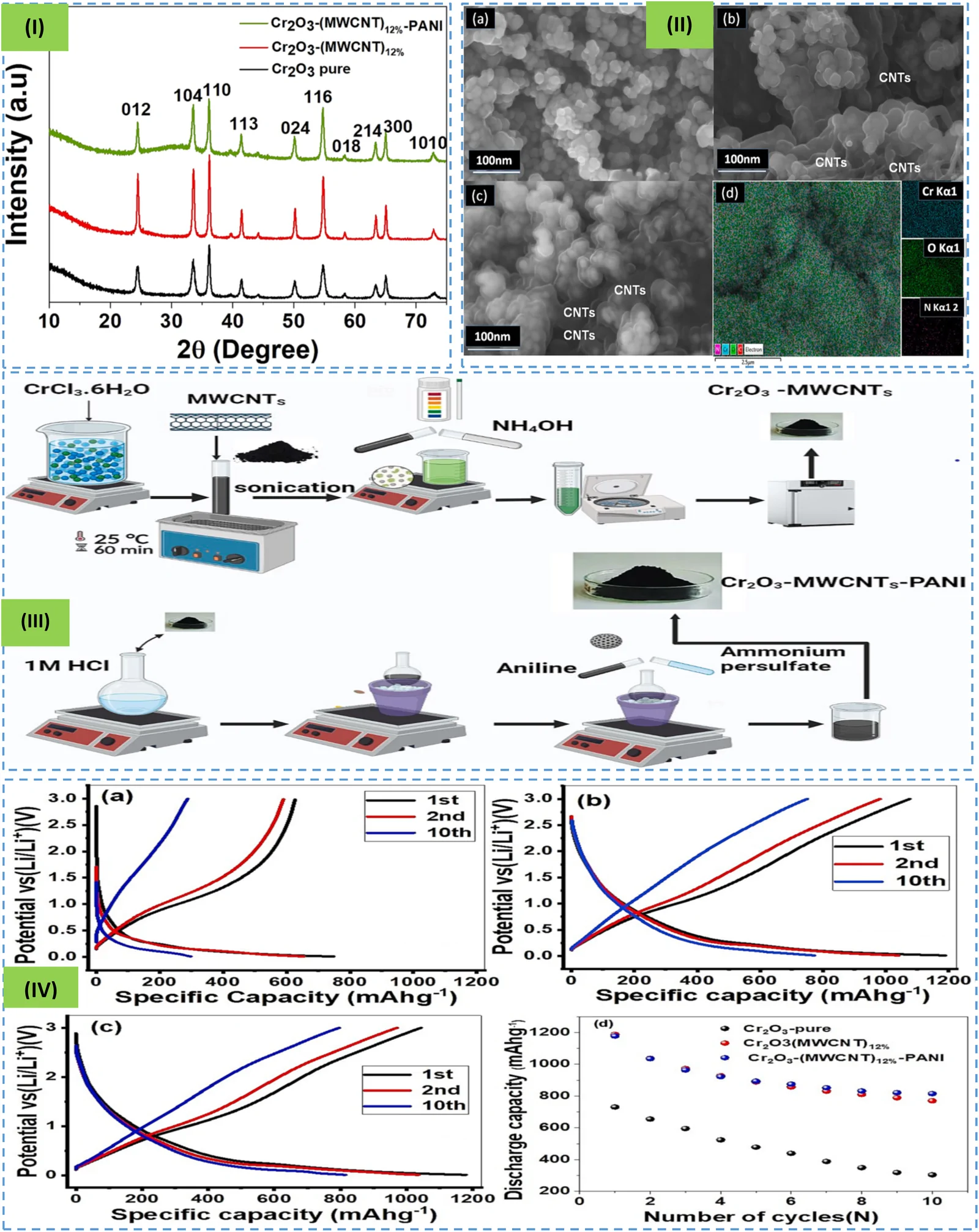
(I) X-ray diffraction patterns of Cr_2_O_3_, Cr_2_O_3_ combined with 12% MWCNTs, and Cr_2_O_3_–MWCNT(12%)–PANI nanocomposites. (II) Field emission scanning electron microscopy (FESEM) images of (a) pure Cr_2_O_3_, (b) Cr_2_O_3_ mixed with 12% MWCNTs, and (c) the Cr_2_O_3_–MWCNT (12%)–PANI hybrid, and (d) elemental mapping (EDX) of the Cr_2_O_3_–MWCNT (12%)–PANI sample. (III) Conceptual diagram illustrating the formation of Cr_2_O_3_–MWCNT (*x*%) and Cr_2_O_3_–MWCNT (*x*%)–PANI nanocomposites. (IV) Electrochemical performance: graphs showing how much charge and discharge occurs for (a) Cr_2_O_3_, (b) Cr_2_O_3_–MWCNT (12%), and (c) Cr_2_O_3_–MWCNT (12%)–PANI, and (d) how stable these materials are over time. Figures reproduced with permission from *Elsevier*.^101^

Mesoporous Cr_2_O_3_/MWCNTs/PANI nanocomposite as a high-performance anode material for RB research was investigated by Laraib Kiran *et al.* (2023).^60^ Synthesizing a Cr_2_O_3_/MWCNT nanocomposite *via* a low-cost co-precipitation method, enhancing conductivity and structural integrity, is shown in Fig. 6(III). Polyaniline (PANI) coating, applied through *in situ* polymerization, improves lithium-ion transport and electron conductivity (Fig. 6(IV)). The Cr_2_O_3_–MWCNTs (12%)–PANI composite achieves a high discharge capacity of 815 mA h g^−1^ at 100 mA g^−1^ with 97.5% coulombic efficiency, showing excellent stability and rate performance.

The XRD analysis results for pure Cr_2_O_3_, Cr_2_O_3_–MWCNTs (12%), and Cr_2_O_3_–MWCNTs (12%)–PANI are illustrated in Fig. 6(I). The calculated sizes of the crystals for these samples are about 23 nm for pure Cr_2_O_3_, 24.1 nm for Cr_2_O_3_-MWCNTs (12%), and 29.4 nm for Cr_2_O_3_–MWCNTs (12%)–PANI, showing a small increase in size after adding MWCNTs and coating with PANI. ESEM analysis indicates that the nanoparticles exhibit a uniform quasi-spherical morphology with noticeable agglomeration among the fine particles. The FESEM image of the Cr_2_O_3_–PANI composite further confirms that Cr_2_O_3_ particles are evenly embedded within the PANI matrix, as illustrated in Fig. 6(II(c)).

### 2.3. Mesocrystal-based MWCNT anodes for RBs

Mesocrystals are ordered superstructures composed of highly oriented nanocrystals sharing a common crystallographic alignment. This unique architecture combines the advantages of single crystals and nanostructured materials, offering high surface area, abundant active sites, and efficient ion transport pathways. When integrated with MWCNTs, mesocrystal-based anodes benefit from enhanced electrical conductivity, improved structural stability, and accelerated charge transfer kinetics, making them promising candidates for high-performance lithium-ion battery applications.

**Fig. 7:**
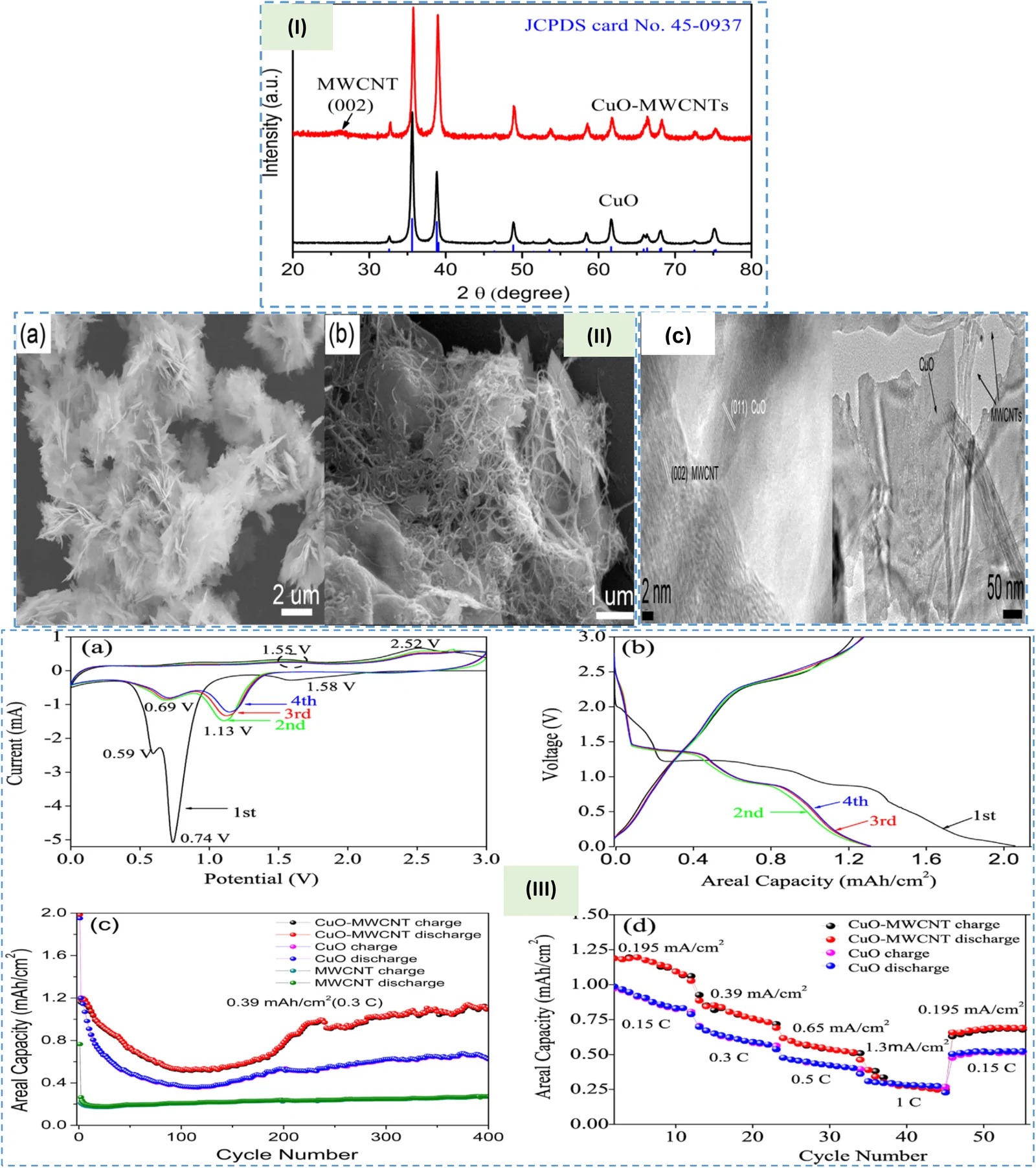
(I) XRD spectra of pure CuO, MWCNTs, and CuO–MWCNT composite materials. (II) Morphological analysis: SEM images of (a) pure CuO and (b) the CuO–MWCNT composite; (c) TEM image of the CuO–MWCNT composite. (III) Electrochemical performance: (a) CV curves and (b) charge–discharge profiles of the CuO–MWCNT anode; (c) cycling stability of CuO, CuO–MWCNT, and MWCNT anodes; (d) comparison of rate capability between CuO and CuO–MWCNT anodes. Figure reproduced from ref. 102 with permission from *Elsevier*. Various metal oxide-based MWCNT composites and their performances are shown in Table 3.^103^

Junmin Xu and colleagues reported a facile and scalable method to synthesize CuO mesocrystal/MWCNT composites *via* precipitation, followed by oriented aggregation.^102^ These composites, when used as anode materials for lithium-ion batteries, showed a high capacity of 1.11 mA h cm^−2^ after 400 cycles at a current of 0.39 mA cm^−2^ and remained stable over time. The excellent performance comes from the combination of defect-rich CuO mesocrystals, which help move Li-ions and handle changes in size, and flexible MWCNTs, which keep the structure stable and maintain electrical connections during use.^102^Fig. 7(I) presents the XRD patterns of CuO and CuO–MWCNT composites, where the weak peak at 26° confirms the presence of MWCNTs. SEM images (Fig. 7(II)) reveal the transformation of flower-like CuO structures into a 3D network architecture after MWCNT incorporation. TEM analysis (Fig. 7(III(f))) further confirms the successful integration of MWCNTs into the CuO structure. As shown in (Fig. 7(III(a))), the first cathodic scan of the CuO–MWCNT electrode shows three clear peaks at about 1.58, 0.74, and 0.59 V, which are related to the creation of the solid solution Li_*x*_CuO and the later change to Cu_2_O. Fig. 7(III(b)) shows the typical charge and discharge curves of the CuO–MWCNT composites obtained at a current density of 0.195 mA cm^−2^ (about 0.15C, where 1C equals 670 mA g^−1^) within a voltage range of 0 to 3.0 V. The cycling performance of the CuO–MWCNT composites and the regular CuO sheets is shown in Fig. 7(III(c)), tested at a current density of 0.390 mA cm^−2^ (about 0.3C) within a voltage range of 0.01 to 3.0 V. Fig. 7(III(d)) also shows how well the pure CuO and CuO–MWCNT electrodes perform at different current levels.

**Table 3:** Electrochemical performance of various anode materials

Anode materials	First cycle discharge (mA h g^−1^)	Coulombic efficiency (%)	Current density (mA g^−1^)
MWCNTs^103^	189.9	96	50
MnO_2_/MWCNTs^103^	200.1	97	50
Fe_3_O_4_/MWCNTs^103^	210.2	97	50
MnO_2_/Fe_3_O_4_/MWCNTs^103^	207.1	98	50
N-Doped graphene/Fe–Fe_3_C^104^	904	24	100
CoO/N-CNTs^105^	1250	83	100
Cage-like CNTs/Si^106,107^	730	68	100
CuO/CNT^108^	NR	56	100
Fe_3_O_4_/C/CNTs^109^	NR	75.7	200
Porous Fe_3_O_4_/C^110^	1796	56.2	100

**Fig. 8:**
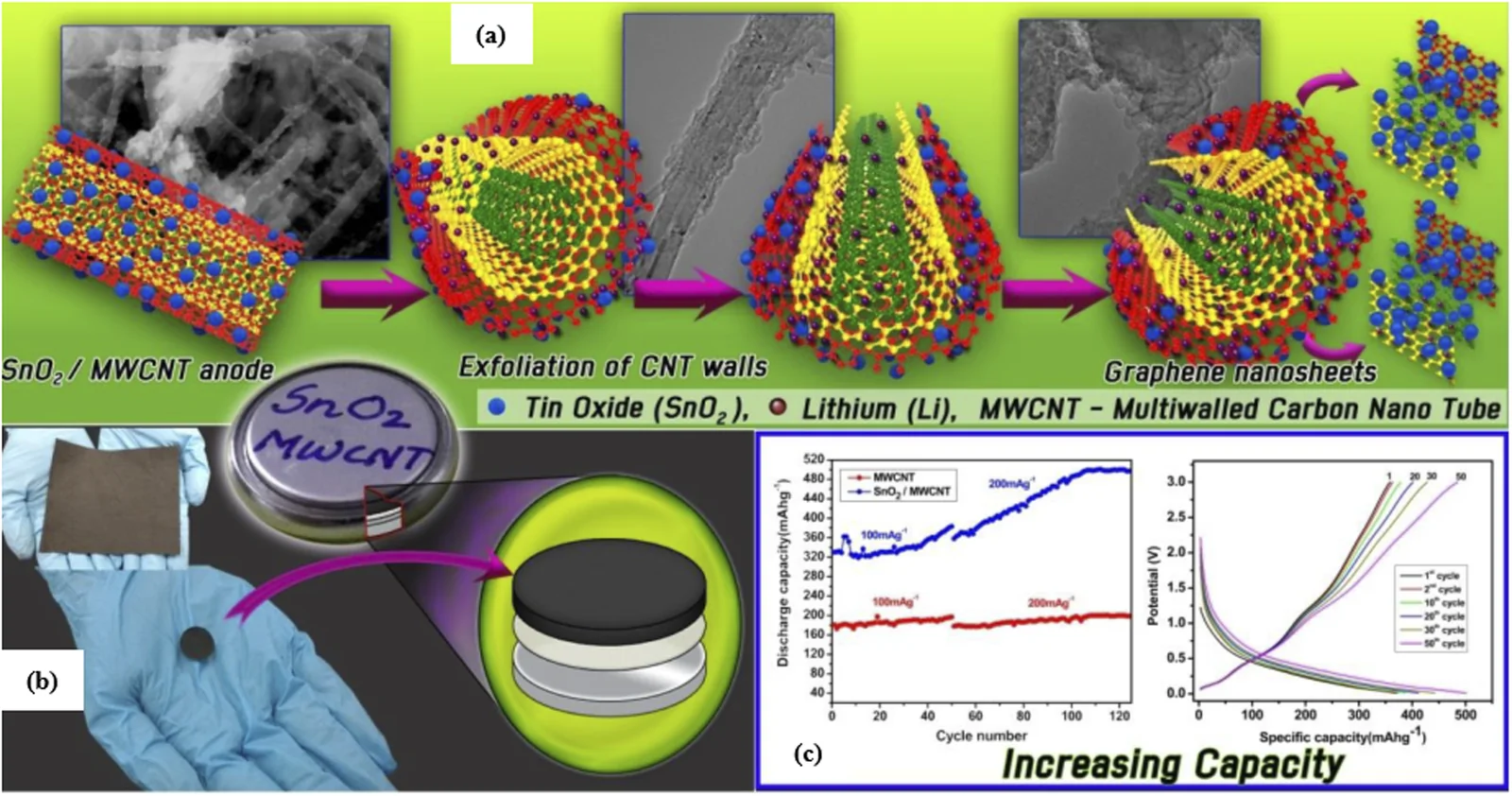
(a) Proposed mechanism illustrating the enhancement of lithium storage capacity in the SnO_2_/MWCNT composite anode. (b) Photograph of the coin-cell assembled using the SnO_2_/MWCNT composite. (c) Electrochemical performance of the composite: discharge capacity *versus* cycle number at current densities of 100 mA g^−1^ (50 cycles) and 200 mA g^−1^ (70 cycles), along with the potential *versus* specific capacity curve at 200 mA g^−1^. Figures adapted with the permission from ref. 111.

Huaixin Liu *et al.* conducted research on electrochemical performance of MnO_2_/Fe_3_O_4_/MWCNTs nanomaterials as anodes for a RB.^103^ The study found that the MnO_2_/Fe_3_O_4_/MWCNTs nanomaterials exhibited superior electrochemical performance as a RB anode, with high initial (207.1 mA h g^−1^) and 70th-cycle (189.5 mA h g^−1^) discharge capacities at 50 mA g^−1^, along with excellent capacity retention (93.3%). The synergy between MnO_2_ and Fe_3_O_6_ nanoparticles enhanced conductivity, stability, and reversibility. The MnO_2_/Fe_3_O_4_/MWCNT composites demonstrate that combining metal oxides with MWCNTs can effectively enhance conductivity, structural stability, and electrochemical performance through synergistic interactions and improved charge transport pathways. Building on these findings, SnO_2_/MWCNT composites have also attracted considerable attention due to their high theoretical capacity and the ability of MWCNTs to alleviate volume expansion during cycling. Elizabeth *et al.* (2015) demonstrated that an *in situ* morphological transformation from MWCNTs to graphene nanosheets (GNS) occurs upon lithium intercalation. This transition results in a significant capacity enhancement of over 50% for SnO_2_/MWCNT composite anodes during cycling in RBs, as illustrated in Fig. 8.^111^

**Fig. 9:**
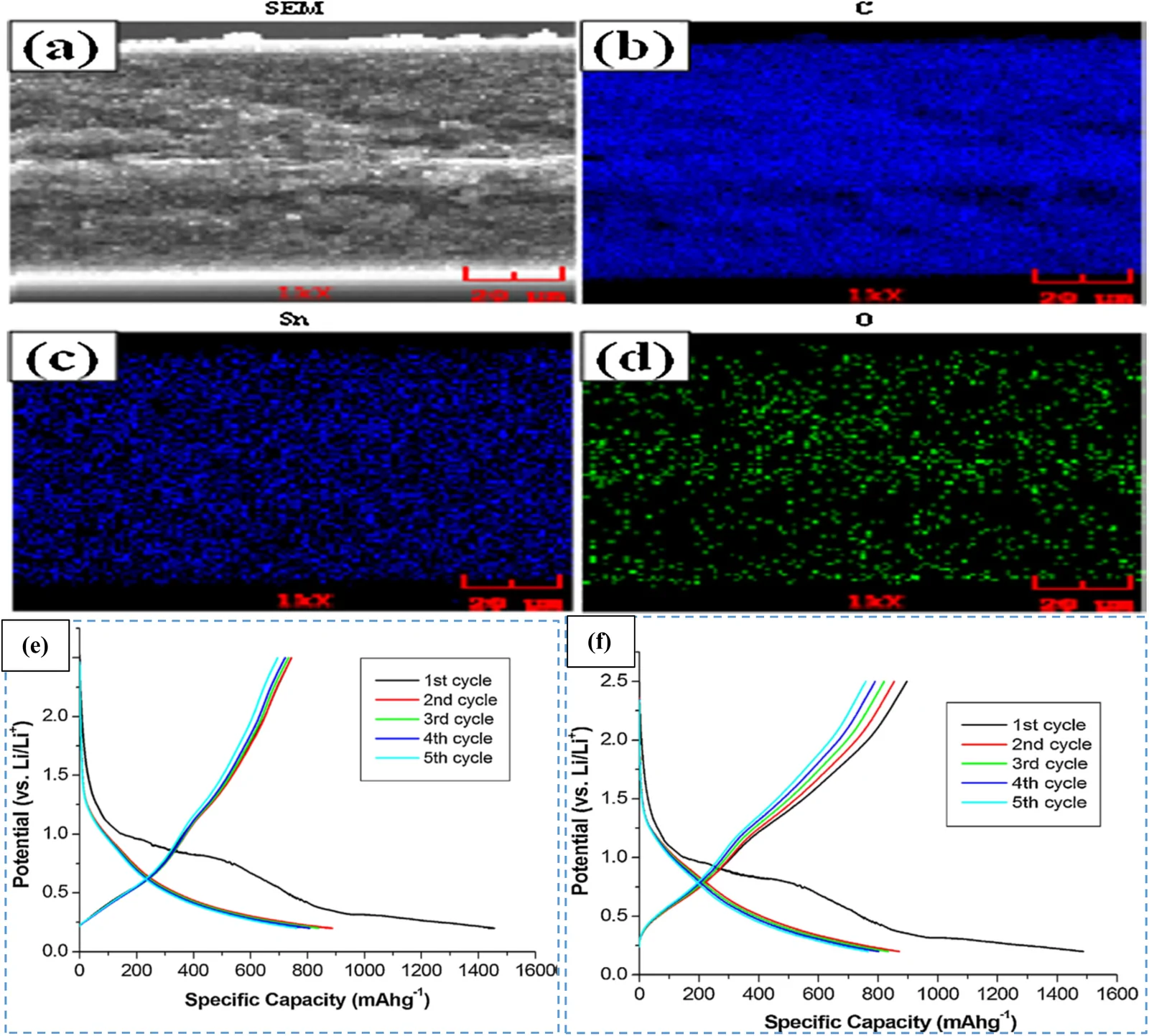
Elemental mapping analysis of (a) SEM image showing the cross-section of the SnO_2_/MWCNT buckypaper composite film. Elemental distribution maps of (b) carbon (C), (c) tin (Sn), and (d) oxygen (O) within the MWCNT buckypaper structure. (e and f) Electrochemical performance of the SnO_2_/MWCNT buckypaper composites: galvanostatic charge–discharge voltage profiles recorded over five cycles within a voltage range of 0.2–2.5 V. Adapted with permission from ref. 112.

SnO_2_/MWCNT nanomaterial anodes synthesized *via* a low-cost sol–gel method demonstrated high capacity and mechanical stability. The MWCNT buckypaper acted as a buffer, preventing structural degradation during cycling.^112^ This nanocomposite anode shows strong potential as a cost-effective anode material for lithium-ion battery applications with further optimization. Elemental mapping analysis and electrochemical performance results of the SnO_2_/MWCNT anode are presented in Fig. 9(A–F).

### 2.4. Silicon-based MWCNT anodes for RBs

Silicon/MWCNT composites exemplify this synergy, where MWCNTs mitigate silicon's volume expansion (>300%) and low conductivity (∼10^−3^ S cm^−1^). A recent study using a triple DC thermal plasma jet system synthesized Si–MWCNT nanocomposites by injecting silicon and MWCNTs separately, achieving uniform anchoring of sub-20 nm silicon nanoparticles on MWCNT surfaces. This method enhanced interfacial contact and lithiation kinetics, though full electrochemical data remain pending.^113^ Another approach developed flexible, binder-free Si/oxidized MWCNT (OCNT) anodes, where chemical reduction (Si/OCNT-C) restored conductivity disrupted by acid treatment, yielding 768 mA h g^−1^ after 150 cycles, 40% higher than thermally reduced counterparts by optimizing dispersion and charge transfer efficiency.^114^

Binder-free architectures further optimize performance by eliminating inactive components. A Cu-grown MWCNT anode exhibited triple the graphite capacity (∼3 × 372 mA h g^−1^) and retained full capacity over 50 cycles at 3C rate, attributed to direct substrate bonding and efficient ion intercalation.^115^ Such designs align with trends toward sustainable synthesis; for instance, thermal plasma systems offer scalable, solvent-free production of metal/MWCNT composites.,^113^ while chemical reduction methods balance conductivity and cost.^114^ Challenges persist in achieving industrial-scale production costs and first-cycle coulombic efficiencies (>90%), but pre-lithiation techniques and hybrid composites (*e.g.*, MWCNT–graphite–metal blends) show promise for bridging lab-scale innovations to commercial viability. By integrating MWCNTs, the mechanical resilience and conductive networks with high capacities of metals, these nanocomposites represent a critical pathway toward next-generation RBs with enhanced energy density and longevity.

Silicon has one of the highest theoretical capacities (∼4200 mA h g^−1^) among anode materials, but it undergoes drastic volume changes (∼300%) during cycling, causing mechanical degradation. MWCNTs act as a flexible matrix that buffers volume expansion and maintains electrical connectivity. Si/MWCNT nanocomposites have also been developed as a transformative solution for RB anodes, because silicon has ultrahigh theoretical capacity (3570 mA h g^−1^) while mitigating its critical limitations through synergistic integration with MWCNTs. Despite the advantages of silicon, its severe volume expansion (>300%) during lithiation, low intrinsic conductivity (∼10^−3^ S cm^−1^), and unstable SEI formation lead to rapid capacity fading and mechanical degradation.^116^ MWCNTs address these issues by providing a conductive 3D scaffold that buffers mechanical stress, enhances electron transport, and prevents silicon nanoparticle aggregation.^116,117^ Recent advancements in synthesis strategies and composite engineering have significantly improved performance, though challenges persist in scalability and long-term cyclability. However, a primary challenge lies in maintaining structural integrity during cycling. Pure silicon particles pulverize under repeated expansion/contraction, causing electrode disintegration. The high tensile strength (up to 63 GPa) of MWCNTs mechanically stabilizes silicon, as demonstrated in freestanding Si/oxidized MWCNT (OCNT) composites.^117^ Acid-treated MWCNTs improve dispersion but reduce conductivity due to disrupted sp^2^ bonds. A recent study in 2024 introduced post-fabrication chemical reduction, restoring conductivity while retaining dispersion benefits. The resulting Si/OCNT-C anode achieved 768 mA h g^−1^ after 150 cycles with a 40% reduction in charge transfer resistance, outperforming its thermally reduced counterparts (543 mA h g^−1^).^118,119^ This approach highlights the importance of balancing dispersion and conductivity through tailored reduction processes.

Another critical issue is optimizing interfacial contact between silicon and MWCNTs. Loose physical mixing often leads to delamination during cycling. Thermal plasma synthesis offers a scalable solution by directly anchoring silicon nanoparticles (<20 nm) onto MWCNT surfaces. By varying injection parameters in a triple DC plasma jet system, researchers achieved uniform Si–MWCNT nanocomposites with enhanced lithiation kinetics, though full electrochemical data remain pending.^116,117^ Covalent bonding strategies, such as nitrogen doping, further strengthen interfaces. Nitrogen-doped SWCNTs accelerate Li-ion diffusion and stabilize silicon expansion. Nitrogen's electron-rich surface improved interfacial charge transfer, while graphene encapsulation provided additional mechanical support, though SWCNT dispersion challenges required novel functionalization techniques.^118^

**Fig. 10:**
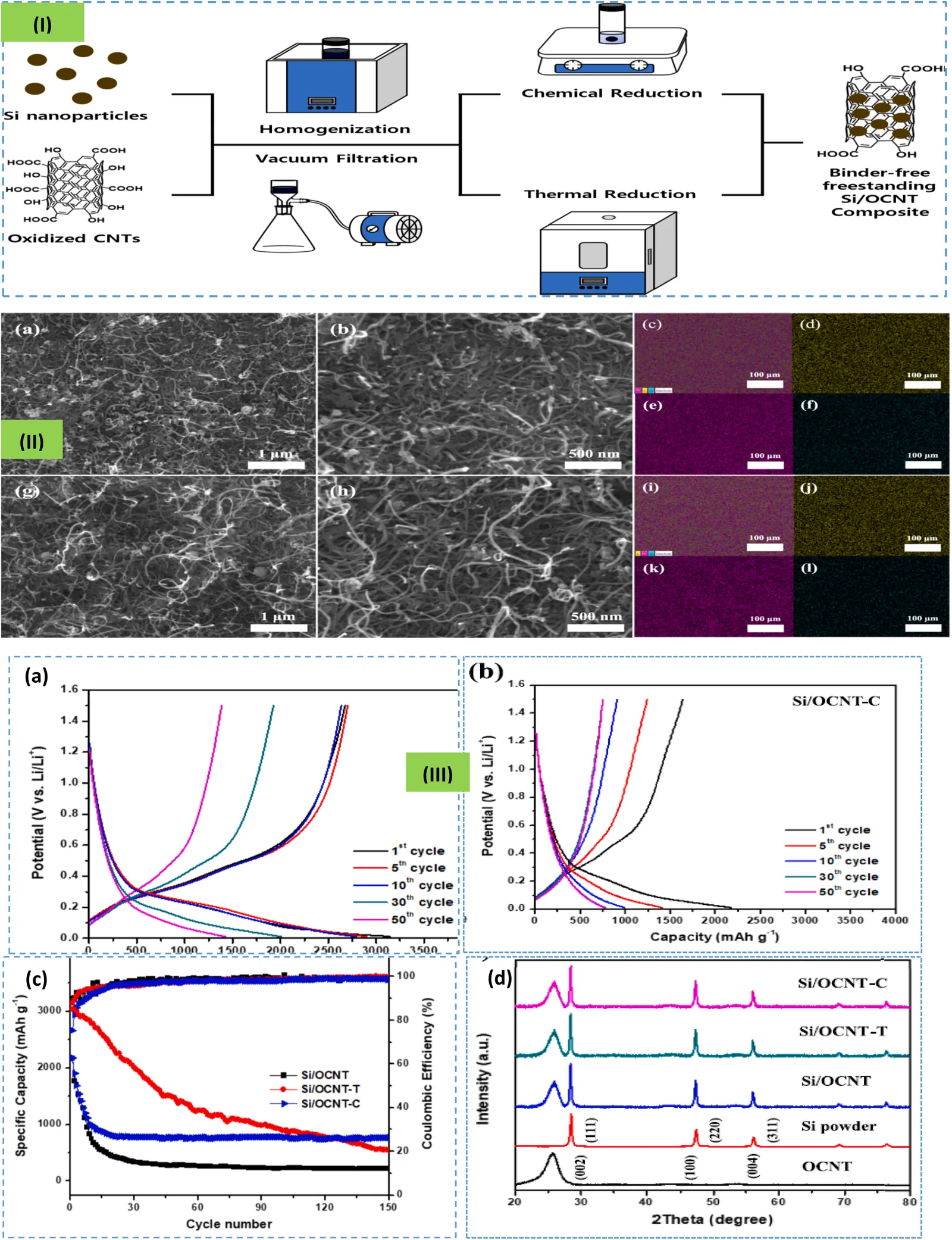
(I) Schematic illustration of the fabrication process for binder-free freestanding Si/OCNT composites. (II) (a and b) Low- and high-magnification images of the Si/OCNT-T composite; (c–f) EDS mapping of the Si/OCNT-T composite; (g and h) low- and high-magnification images of the Si/OCNT-C composite; (i–l) EDS mapping of the Si/OCNT-C composite. (III) (a) Charge/discharge profiles of Si/OCNT-T and (b) Si/OCNT-C composite anodes at 0.2 C. (c) Cycling performance and coulombic efficiency of Si/OCNT, Si/OCNT-T, and Si/OCNT-C anodes at 0.2 C. (d) XRD patterns of composites. Adapted with permission ref. 115. The Si-coated MWCNT tissue composite anode was produced by magnetron sputtering and used as a flexible freestanding anode for improved RBs, according to Huang *et al.* (2024). After the first ten creation cycles, this composite anode permits up to 94% coulombic efficiency and a notable increase in gravimetric capacity from 109 to 290 mA h g^−1^ in comparison to uncoated MWCNT tissue (Fig. 11).^122^

Jung-Soo Kim *et al.* (2025) researched a flexible, binder-free silicon/oxidized carbon nanotube anode for lithium-ion batteries with improved electrochemical performance *via* chemical reduction.^116^ The tendency of MWCNTs to aggregate due to π–π interactions requires acid treatment to improve dispersion, which unfortunately lowers electrical conductivity. To resolve this, a post-fabrication chemical reduction step was introduced in Si/oxidized MWCNT (OCNT) composite anodes. The binder-free, freestanding Si/OCNT-C composite was prepared through a process illustrated schematically in Fig. 10(I). The developed anode showed lower charge transfer resistance, strong bending stability, and retained a discharge capacity of 768 mA h g^−1^ after 150 cycles as shown in Fig. 10(III(a)–(c)). These results demonstrate the importance of chemical reduction in boosting the performance of Si/OCNT anodes for advanced lithium-ion batteries. XRD analysis (Fig. 10(III(d))) shows that Si/OCNT, Si/OCNT-T, and Si/OCNT-C composites retain the characteristic Si and OCNT peaks, indicating that thermal and chemical treatments do not alter the crystalline structure of Si. SEM and EDS mapping (Fig. 10(II)) reveal that Si/OCNT-T exhibits a denser structure with reduced porosity, which may influence thickness, electrochemical performance, and mechanical stability. In contrast, Si/OCNT-C maintains a uniform 3D CNT network similar to Si/OCNT, suggesting minimal impact from the chemical reduction process. Fig. 10(III(a)–(c)) illustrates the charge and discharge patterns, as well as the cycling performance and coulombic efficiency of Si/OCNT-based anodes. Si/OCNT-T and Si/OCNT-C exhibited initial capacities of 3145 and 2176 mA h g^−1^, respectively, with Si/OCNT-T demonstrating superior performance attributed to enhanced SEI production and effective volume expansion management. The initial coulombic efficiencies were 90% for Si/OCNT, 85% for Si/OCNT-T, and 75.4% for Si/OCNT-C. After 150 cycles, Si/OCNT-C exhibited the maximum capacity (768 mA h g^−1^), followed by Si/OCNT-T (543 mA h g^−1^) and Si/OCNT (218 mA h g^−1^). Si/OCNT-C exhibited greater stability owing to improved conductivity from chemical reduction, but Si/OCNT-T experienced capacity degradation due to inadequate mechanical flexibility and instability of the solid electrolyte interphase (SEI).^120,121^ This work emphasizes the important function of chemical reduction in improving the electrochemical performance of Si/OCNT composite anodes, therefore offering important new perspectives for improving RB performance.

**Fig. 11:**
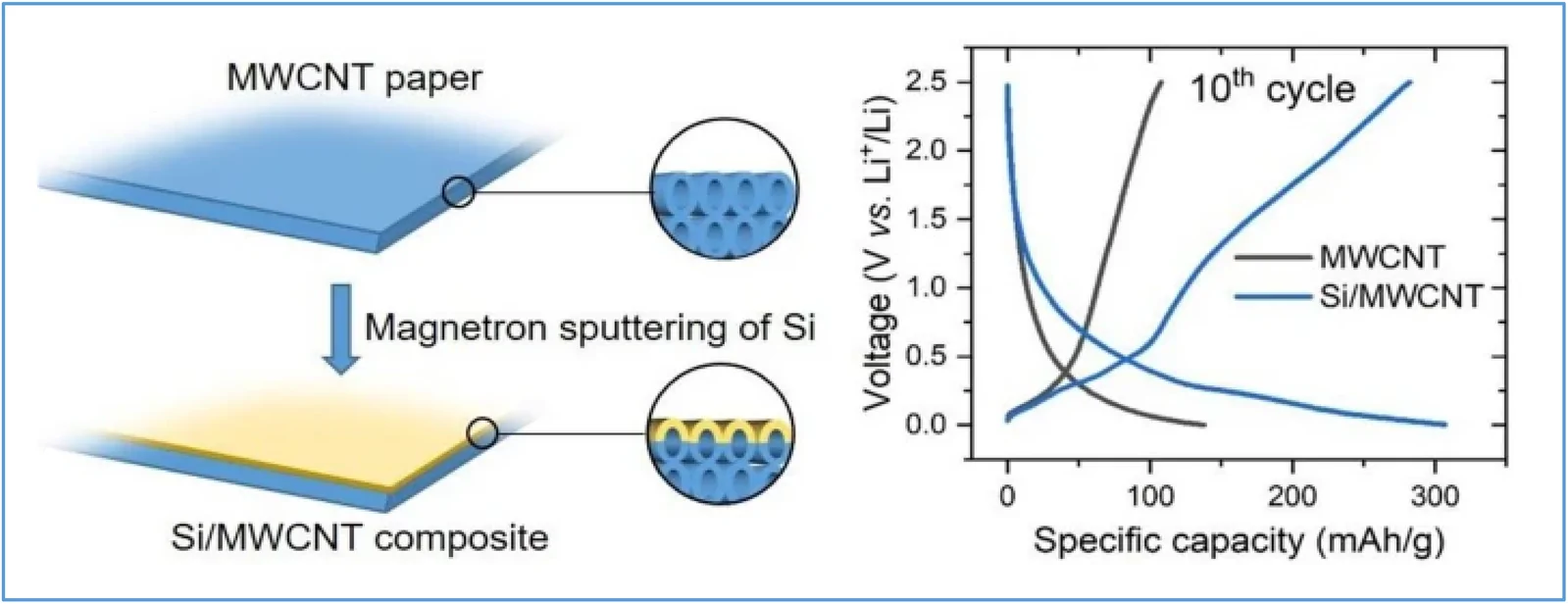
Si-Coated MWCNT tissue composite material fabricated *via* magnetron sputtering for application as a flexible freestanding anode for advanced RBs.^122^

Recent designs emphasize binder-free architectures to maximize energy density. Flexible Si/MWCNT papers fabricated *via* ultrasonication and pressure filtration eliminated traditional copper current collectors, achieving 1170 mA h g^−1^ at 100 mA g^−1^. However, areal capacities (<5 mA h cm^−2^) and initial coulombic efficiencies (75–85%) lag behind those of commercial graphite. Pre-lithiation techniques using stabilized lithium metal powder (SLMP) and machine learning-driven compositional optimization are being explored to address these gaps. Hybrid composites blending MWCNTs with graphene or graphite show promise for balancing capacity and stability, as seen in a nitrogen-doped SWCNT–graphene–silicon system.^119^

Finally, it can be concluded that MWCNTs significantly improve the electrochemical performance of Si-based anodes by providing highly conductive pathways for rapid electron transport and forming a flexible interconnected network that buffers the severe volume expansion of Si during lithiation/delithiation. This conductive and mechanically robust framework helps maintain structural integrity, prevents particle pulverization, reduces electrode degradation, and enhances cycling stability and rate capability.

### 2.5. Silicon doped carbon-based MWCNT anodes for RBs

**Fig. 12:**
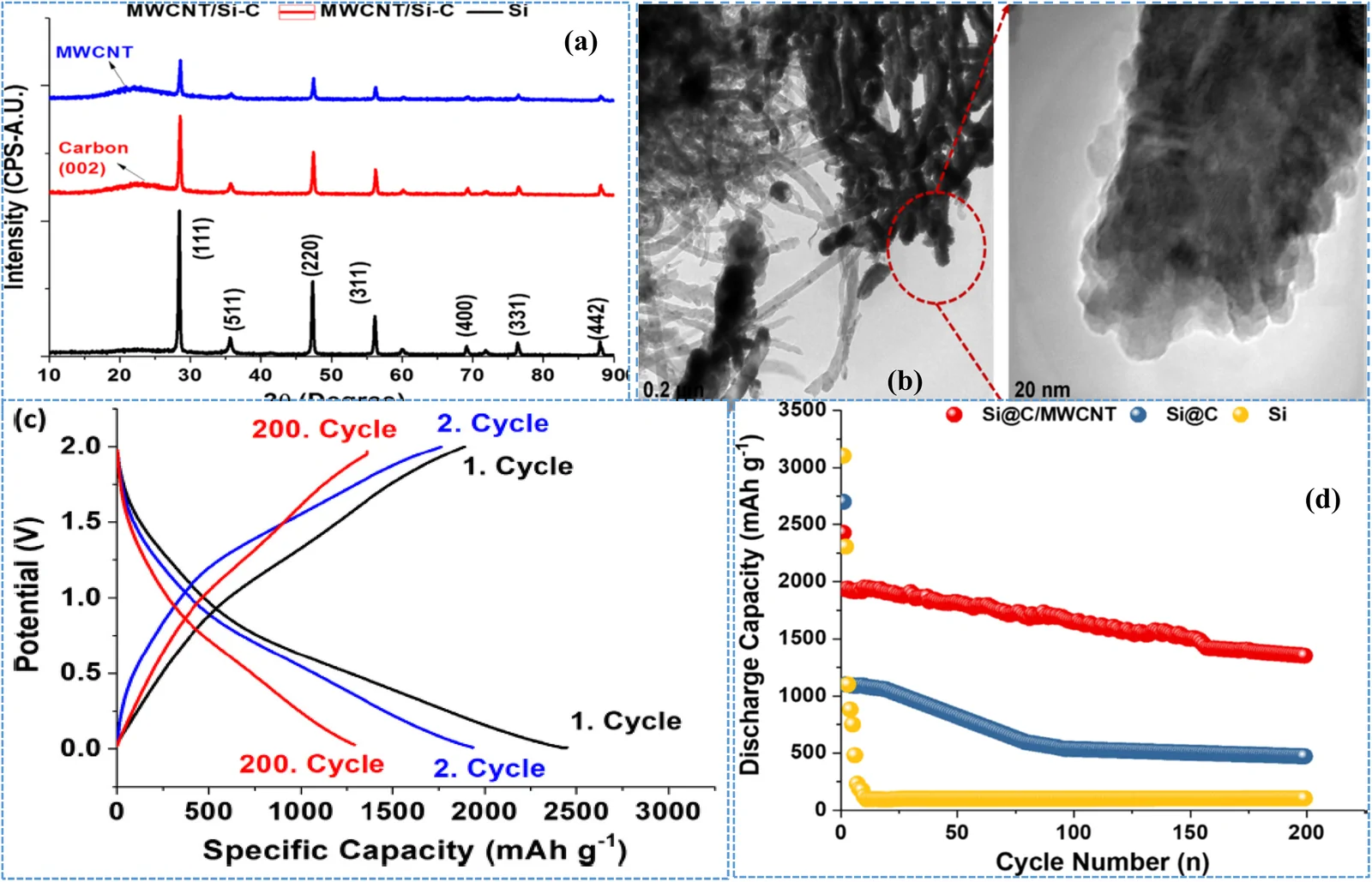
(a) XRD patterns of Si, Si@C, and Si@C/MWCNT anode materials, showing structural characteristics. (b) TEM image illustrating the morphology of the Si@C/MWCNT freestanding electrode. (c) Charge–discharge profiles of the Si@C/MWCNT electrode under galvanostatic cycling. (d) Comparison of specific discharge capacity *versus* cycle number for Si, Si@C, and Si@C/MWCNT electrodes. Figure reinterpreted from ref. 123.

**Fig. 13:**
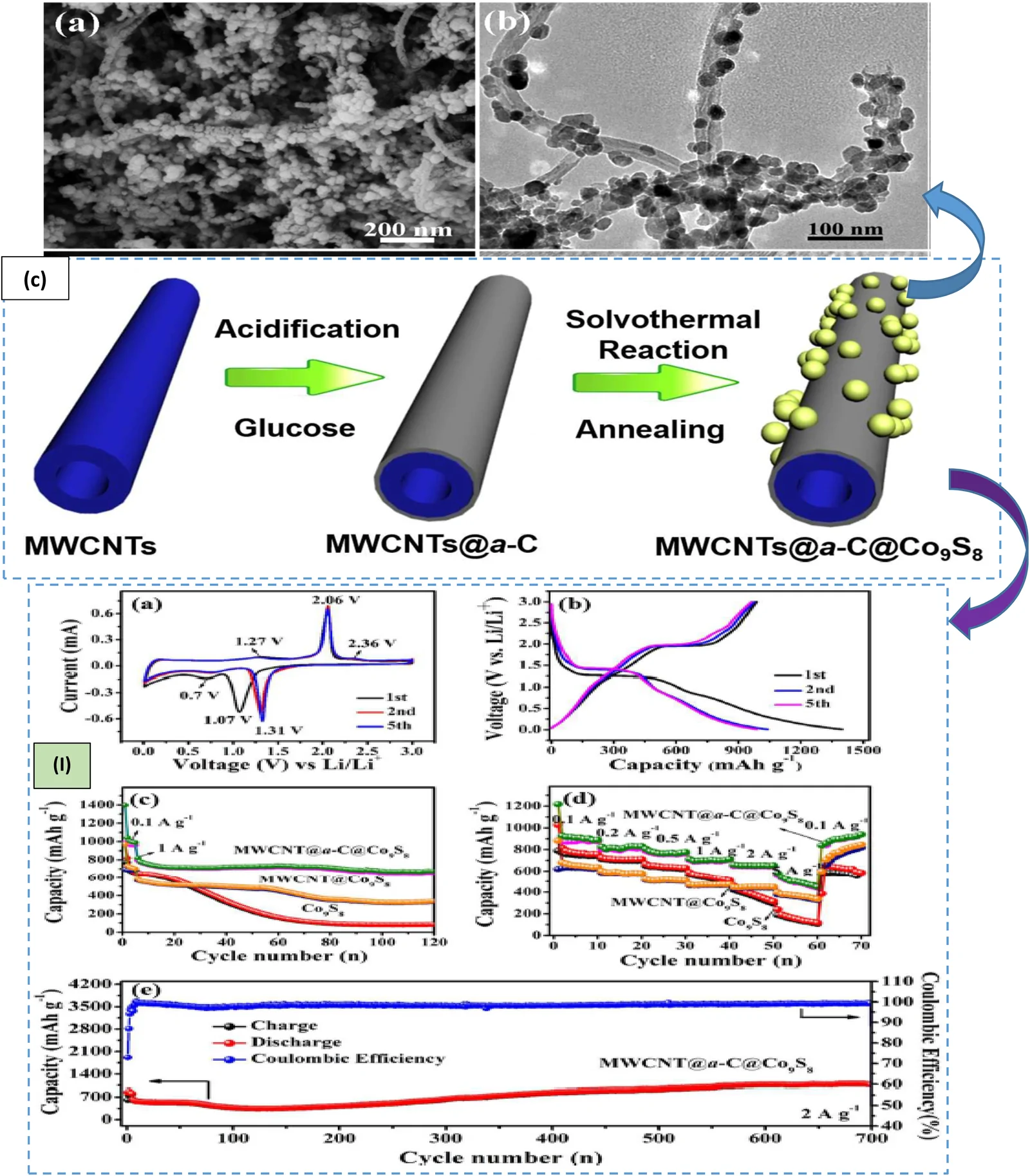
(a) SEM and (b) TEM images of MWCNT@a-C@Co_9_S_8_ nanocomposites. (c) Schematic illustration of the synthesis process for MWCNT@a-C@Co_9_S_8_. (I) Electrochemical characterization: (a) cyclic voltammetry curves and (b) charge–discharge profiles of MWCNT@a-C@Co_9_S_8_ electrodes. (c) Cycling stability and (d) rate performance comparison among MWCNT@a-C@Co_9_S_8_, MWCNT@Co_9_S_8_, and pure Co_9_S_8_ electrodes. (e) Long-term cycling performance of MWCNT@a-C@Co_9_S_8_ at 2 A g^−1^ over 700 cycles. Reproduced with permission from ref. 131.

H. Akbulut *et al.* (2018) developed a free-standing Si@C/MWCNT nanocomposite *via* vacuum filtration.^123^ The composite exhibited a stable capacity of 1290 mA h g^−1^ after 200 cycles, attributed to the conductive network and structural support of MWCNTs, which mitigated Si volume changes and improved electrochemical performance for energy storage applications.^123^Fig. 12(a) presents the XRD patterns of Si, Si@C, and MWCNT/Si@C composites, where the broad peak at 26.5° confirms the carbon structure and indicates the formation of a network surrounding the Si particles. TEM analysis (Fig. 12(b)) further shows that Si@C nanoparticles are attached to the MWCNT surfaces through defect sites and van der Waals interactions.^124^ After vacuum filtration, the Si@C particles are sealed within the buckypaper, enhancing conductivity and buffering mechanical stress from Si expansion during cycling. The charge and discharge graphs for MWCNT/Si@C nanocomposites at 1C showed high initial discharge capacities of 2702 and 2428 mA h g^−1^, and a stable capacity of 1353 mA h g^−1^ after 200 cycles. This excellent performance is attributed to the novel freestanding electrode architecture achieved *via* vacuum filtration. Fig. 13(d) shows cycling performance at 1C for 200 cycles, where bare Si electrodes exhibited rapid capacity fade due to volumetric expansion. In contrast, Si@C and MWCNT/Si@C composites retained 451 mA h g^−1^ and 1353 mA h g^−1^, respectively. The better stability of MWCNT/Si@C is due to its 3D conductive network, which helps manage volume changes and improves Li-ion movement.

### 2.6. Metal sulfide-based MWCNT anodes for RBs

Metal sulfide/MWCNT composites have emerged as promising anode materials for RBs, addressing challenges like poor conductivity and volume expansion through synergistic nanostructuring. Metal sulfides such as MoS_2_, CuS, and NiS offer high capacity but face issues like poor conductivity and volume expansion. MWCNTs improve their electrochemical performance by enhancing electron transport and structural stability. Recent advancements highlight innovative synthesis strategies and performance enhancements. For example, a covalently coupled ZnS/CNT composite utilized amide linkages to anchor ZnS nanoparticles (∼10 nm) onto CNTs, achieving 333 mA h g^−1^ at 2 A g^−1^ over 4000 cycles in RBs due to enhanced electron/ion transfer and structural stability.^125^ Similarly, CuCo_2_S_4_/MWCNT composites demonstrated exceptional rate capability, delivering 1300 mA h g^−1^ at 500 mA g^−1^ for 200 cycles, attributed to MWCNTs mitigating particle aggregation and improving conductivity.^126^ The techniques of direct growth have also proven quite effective. Cylindrical MoS_2_ nanostructures grown on CNTs *via* microwave irradiation exhibited superior cycling stability, leveraging intimate contact between MoS_2_ sheets and CNTs to prevent agglomeration and enhance Li^+^ diffusion.^127^ For NiS_2_@MWCNTs, the 3D conductive network facilitated pseudocapacitive charge storage, yielding high-capacity retention in sodium-ion batteries (SIBs), and a mechanism potentially transferable to RBs.^128^ Recent work on Sb_2_S_3_/MWCNTs highlighted interfacial covalent bonds (C–S and C–O–Sb) that stabilized the structure during sodiation, achieving 548.7 mA h g^−1^ at 100 mA g^−1^ over 100 cycles in SIBs, with implications for RB applications.^129^

These composites utilize the high conductivity and mechanical flexibility of MWCNTs to buffer volume changes and accelerate ion/electron transport. Covalent bonding strategies, such as those in ZnS/CNT and Sb_2_S_3_/MWCNT systems, ensure robust interfacial contact, critical for long-term cyclability. The integration of pseudocapacitive behavior, as seen in NiS_2_@MWCNTs, further enhances rate performance. Recent trends emphasize sustainable approaches, like using natural stibnite for Sb_2_S_3_ synthesis, combined with nanostructuring to optimize active material utilization.^130^ Collectively, these advancements underscore the potential of metal sulfide/MWCNT composites in developing high-energy, durable RB anodes, with ongoing research focused on scaling production and optimizing interfacial engineering for commercial viability.

A one-dimensional MWCNT@*a*-C@Co_9_S_8_ nanocomposite has been prepared *via* a facile solvothermal reaction followed by a calcination process (Fig. 13(c)) by Zhou and his coauthor.^131^ The amorphous carbon layer between Co_9_S_8_ and MWCNTs acts as a linker to increase the loading of sulfides on MWCNTs. The SEM and TEM images in Fig. 13(a) and (b) show an interconnected network of Co_9_S_8_-coated MWCNTs encapsulated in amorphous carbon. Fig. 13(I(a)–(e)) demonstrates the excellent electrochemical performance of the fabricated anode. When evaluated as an anode material for 10 RBs, the MWCNT@a-C@Co_9_S_8_ nanocomposite shows the advantages of high capacity and long life, superior to Co_9_S_8_ nanoparticles and MWCNT@Co_9_S_8_ nanocomposites. The reversible capacity could be retained at 662 mA h g^−1^ after 120 cycles at 1 A g^−1^. The efficient synthesis and excellent performances of this nanocomposite offer numerous opportunities for other sulfides as a new anode for RBs.

Overall, metal sulfide/MWCNT composites improve anode performance by combining high-capacity sulfides with conductive MWCNT networks, which enhance electron/ion transport and buffer volume changes during cycling. Strong interfacial engineering, including covalent bonding and amorphous carbon layers, further stabilizes the structure and improves cycling durability. Overall, hierarchical MWCNT-based architectures enable significantly enhanced capacity, rate performance, and long-term stability in sulfide anodes.

## 3. Future outlook

Despite their promising electrochemical performance and future potential, MWCNT-based nanomaterials for RB anodes have numerous limitations. Uniform dispersion and close contact between MWCNTs and active materials (*e.g.*, SnO_2_, Si, or metal oxides) are crucial for efficient electron transport and structural integrity during charge/discharge cycles.^132^ Nanoparticle agglomeration and poor interface contact can drastically impair electrode performance and cycling life. Post-treatment chemical reduction restores conductivity, complicating manufacture.^132^

**Fig. 14:**
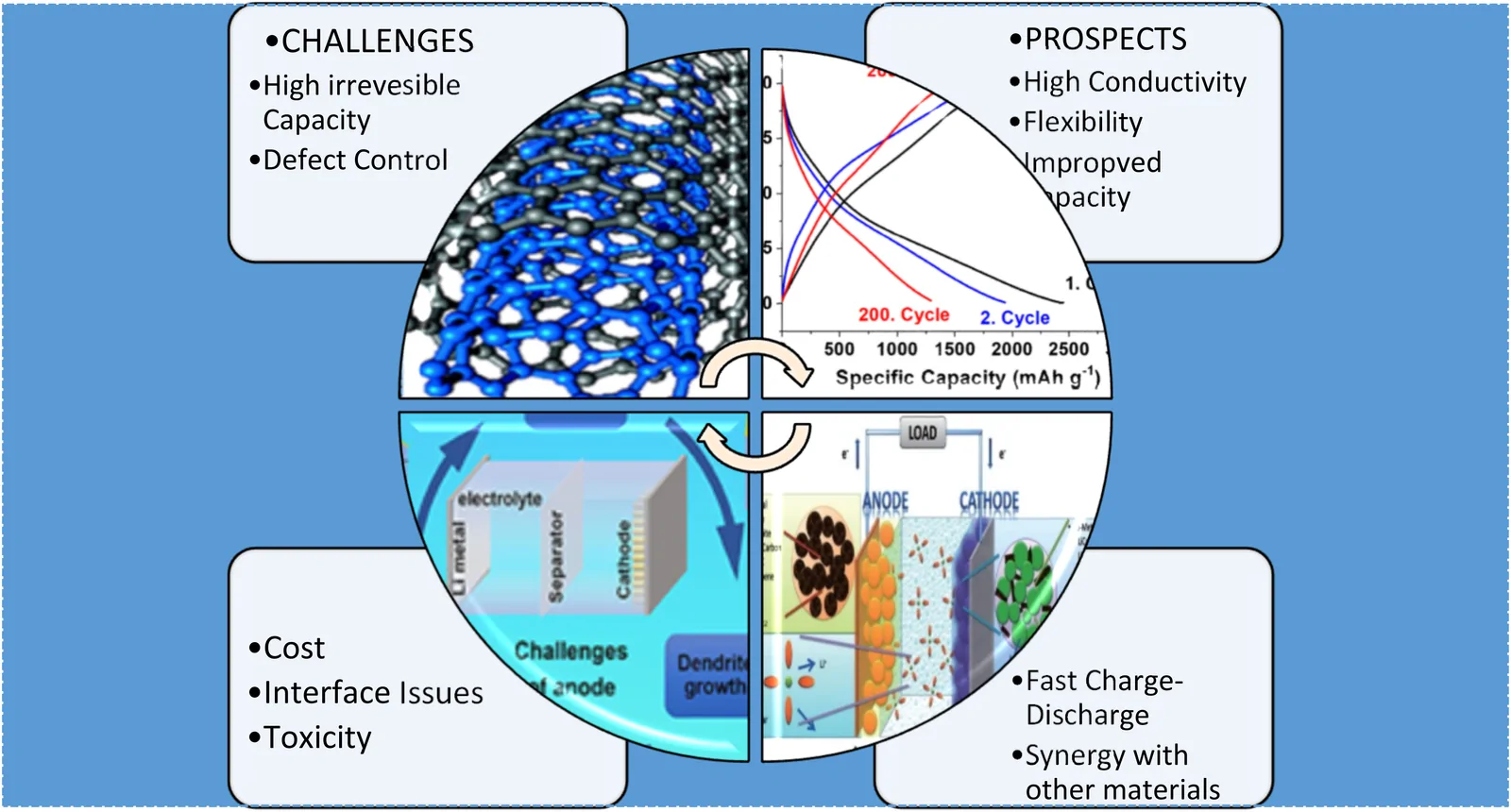
Major challenges and prospects of MWCNT-based anode nanomaterials for RBs.

Although MWCNTs offer excellent electrical conductivity and mechanical flexibility, their expensive production, purification, and processing costs limit their commercial use. Chemical vapor deposition, sol–gel, and hydrothermal synthesis for nanocomposites are difficult, time-consuming, and unscalable, limiting industrial utilization.^133–136^ Due to the SEI layer and irreversible lithium consumption, these composites have low initial coulombic efficiency, especially when combined with high-capacity materials like silicon or transition metal oxides. The thermal stability and degradation processes of MWCNT-based anodes after repeated cycling and diverse operational conditions need more study. CNT handling, toxicity, and disposal pose environmental and health risks that require risk assessment and regulation. Scalable, cost-effective, and environmentally friendly synthesis techniques are needed to generate high-performance MWCNT-based nanocomposites. MWCNTs functionalized with oxygen- or nitrogen-containing groups can improve dispersion and bonding with active materials, while hybrid structures incorporating graphene, amorphous carbon, or conducting polymers may boost conductivity, mechanical strength, and capacity retention.^123^ Coatings or artificial SEI layers can increase ICE and cycle stability. Fig. 14 shows difficulties and opportunities of MWCNT-based RB anode materials.

Future investigations ought to concentrate on developing hybrid architectures that integrate MWCNTs with high-capacity materials such as silicon or metal oxides, utilizing the mechanical resilience of nanotubes to accommodate volume fluctuations while improving Li-ion storage.^132,133^ Creating scalable, economical synthesis techniques, such as roll-to-roll chemical vapor deposition or solution-based processing, may facilitate the transition from laboratory advances to industrial implementation.^132,136^ Advanced doping techniques, including nitrogen or boron functionalization, may enhance conductivity and surface reactivity. Designing electrolytes to align with high-voltage cathodes and incorporating thermally stable elements such as hexagonal boron nitride coatings may improve full-cell performance and safety.^134,135^*In situ* characterization methods will be essential for elucidating degradation mechanisms and enhancing interfacial interactions. Ultimately, the development of mechanically flexible, lightweight MWCNT-based anodes could facilitate applications in wearable electronics and electric vehicles, contingent upon the systematic resolution of these issues.^132,136^

## 4. Conclusions

The performance of RBs has been significantly enhanced by recent developments in MWCNT-based anode nanomaterials. MWCNT-based materials are better than conventional graphite anodes for storing RBs and maintaining their charge stability due to some special advantages. MWCNTs have high mechanical strength, electrical conductivity, vast surface area, and structural stability, which are some of these advantages. Additionally, innovative nanostructure designs like nanocomposites and surface modifications have improved their electrochemical performance, enabling greater reversible capacities and better rate capabilities. Recent findings reveal that there is considerable promise for improving the electrochemical performance of RBs by the integration of metal oxides with MWCNT-based nanostructure anodes. The Si/MWCNT core–shell anode exhibited a high specific capacity of 3250 mA h g^−1^, while the SiO_2_@MWCNT composite achieved 936 mA h g^−1^, highlighting the importance of structural engineering in performance optimization. The Si/MWCNT core–shell anode exhibited a high specific capacity of 3250 mA h g^−1^, while the SiO_2_@MWCNT composite achieved 936 mA h g^−1^, highlighting the importance of structural engineering in performance optimization. Similarly, the MnO_2_/Fe_3_O_4_/MWCNTs hybrid and the mesoporous Cr_2_O_3_/MWCNTs/PANI nanomaterials showed excellent electrochemical properties, confirming the efficiency of MWCNT-based materials in creating high-performance anode materials for next-generation RBs. The conductive network generated by MWCNTs allows for rapid electron transit, while its flexible shape can buffer mechanical loads and reduce electrode pulverization, hence improving electrode structural integrity during cycling. Furthermore, the synergistic interaction of MWCNTs and active materials improves the reversible capacity and total energy density of batteries. Despite many promising experimental results showing stable capacities exceeding 1000 mA h g^−1^ under certain conditions, challenges remain in cost, defect control, and scalability for commercial applications. Nonetheless, MWCNTs continue to be a focal point in research for high-efficiency RBs as anodes because they provide a stable conductive network, facilitate Li-ion diffusion, and help accommodate volume changes during cycling. Continued innovation in material synthesis and composite engineering holds promise to overcome current limitations, potentially making MWCNT-based anodes key components in the next generation of high-performance lithium-ion batteries with greater energy density and durability.

Future directions for MWCNT-based anode nanomaterials in high-efficiency RBs should focus on developing cost-effective and environmentally friendly synthesis methods to overcome current production challenges. Innovations in scalable manufacturing techniques and eco-friendly processes will be critical to facilitate wider commercial adoption. Additionally, integrating MWCNTs with other high-capacity materials, such as silicon or conversion-reaction-type compounds, can potentially enhance energy density and cycling stability by mitigating volume expansion and improving conductivity. Research should also advance the engineering of hybrid composites and surface modifications to boost Li-ion diffusion and electrode durability further. Developing scalable, cost-effective synthesis methods for MWCNT-based anodes will be essential for commercial application advancement. Given the growing demand for electric vehicles and renewable energy storage, optimizing MWCNT anodes for fast charging, high-capacity retention, and safety is essential. Exploring synergies with solid-state electrolytes and AI-driven battery management systems could represent promising directions to improve overall battery performance and lifespan. Lastly, addressing environmental and regulatory concerns related to nanomaterial toxicity and sustainability will be vital for market growth and acceptance.

## Author contributions

Yanlu lv: conceptualization, review & editing. Md. Saiful Islam: conceptualization, supervision, revision. Ghanshyam G. Tejani: review & editing. Feng lin: writing – original draft, formal analysis, data curation. M. A. M. Mohd Idrus: revision, writing and editing.

## Conflicts of interest

The authors declare no competing financial interests or personal relationships.

## Data Availability

This article is a review and does not involve the generation of new primary data. All data, figures, and information discussed in this review are derived from previously published studies, which are openly available in the public domain or accessible through cited literature.

## References

[cit1] Shen K. X., Zhang Z. S., Wang S. F., Ru Q., Zhao L. Z., Sun L. F., Hou X. H., Chen F. M. (2020). Cucumber-shaped construction combining bismuth nanoparticles with carbon nanofiber networks as a binder-free and freestanding anode for Li-ion batteries. Energy Fuels.

[cit2] Liu R. R., Hou L. L., Yue G. C., Li H. K., Zhang J. S., Liu J., Miao B. B., Wang N., Bai J., Cui Z. M., Liu T. X., Zhao Y. (2022). Progress of fabrication and applications of electrospun hierarchically porous nanofibers. Adv. Fiber Mater..

[cit3] Gao J., Shi S.-Q., Li H. (2016). Brief overview of electrochemical potential in lithium ion batteries. Chin. Phys. B.

[cit4] IDTechEx , Advanced Li-ion Batteries 2025–2035: Technologies, Players, Markets, Forecasts, accessed 10 May 2025

[cit5] Chang H., Wu Y. R., Han X., Yi T. F. (2021). Recent developments in advanced anode materials for lithium-ion batteries. Energy Mater..

[cit6] Zhang D., Song Z., Chen Y., Liu P., Gu R., Miao L., Liu M. (2025). Ultralow-Lattice-Mismatched Near-Zero-Strain Zn (0002) Anodes for Stable Zinc Metal Batteries. Angew. Chem., Int. Ed..

[cit7] Liu P., Song Z., Huang Q., Xu Z., Lv Y., Gan L., Liu M. (2026). Tri (Thiophene-Nitrile) Covalent Organic Frameworks With Low HOMO/LUMO Donor/Acceptor for High-Potential-Plateau Zinc Batteries. Angew. Chem., Int. Ed..

[cit8] Guo K., Lv Y., Song Z., Gan L., Liu M. (2026). Positioning versatile inorganic cathode materials in the aqueous zinc-ion battery landscape. Chem. Sci..

[cit9] Orangi S., Manjong N., Clos D. P., Usai L., Burheim O. S., Strømman A. H. (2024). Historical and prospective lithium-ion battery cost trajectories from a bottom-up production modeling perspective. J. Energy Storage.

[cit10] Yuan G., Liu D., Feng X., Zhang Y. (2021). ChemPlusChem.

[cit11] Osiak M., Geaney H., Armstrong E., O'Dwyer C. (2014). Structuring materials for lithium-ion batteries: advancements in nanomaterial structure, composition, and defined assembly on cell performance. J. Mater. Chem. A.

[cit12] Hammouche A., Karden E., De Doncker R. W. (2004). Monitoring state-of-charge of Ni–MH and Ni–Cd batteries using impedance spectroscopy. J. Power Sources.

[cit13] Kim K. H., Moon D. B., Jo M. H., Ahn H. J. (2024). Carbon nanotube-interlocked Si/CNF self-supporting electrode using continuable spraying architecture system for flexible lithium-ion batteries. Appl. Surf. Sci..

[cit14] Kim Y., Song W., Lee S. Y., Jeon C., Jung W., Kim M., Park C. Y. (2011). Low-temperature synthesis of graphene on nickel foil by microwave plasma chemical vapor deposition. Appl. Phys. Lett..

[cit15] Xie J., Yin J., Xu L., Ahmed A. (2024). Nanostructured anode materials for high-performance lithium-ion batteries. J. Alloys Compd..

[cit16] Yang Z. (2018). *et al.*, Enhancement in thermoelectric properties of Te-embedded Bi_2_Te_3_ by preferential phonon scattering in heterostructure interface. Nano Energy.

[cit17] Zheng G. (2014). *et al.*, Interconnected hollow carbon nanospheres for stable lithium metal anodes. Nat. Nanotechnol..

[cit18] Zhu J. (2020). *et al.*, AlF_3_-modified anode-electrolyte interface for effective Na dendrites restriction in NASICON-based solid-state electrolyte. Energy Storage Mater..

[cit19] Khazaeli A., Montazeri A., Aghamohammadi H., Eslami-Farsani R. (2025). Holey Graphene-Silicon Nanocomposites as Superior Anodes for Lithium-Ion Batteries with Excellent Rate Performance and Cyclic Stability. Mater. Chem. Phys..

[cit20] Aghamohammadi H., Hassanzadeh N., Eslami-Farsani R. (2021). A review study on the recent advances in developing the heteroatom-doped graphene and porous graphene as superior anode materials for Li-ion batteries. Ceram. Int..

[cit21] Aghamohammadi H., Eslami-Farsani R., Oskouei H. I. (2024). Electrochemical performance of TiNb_2_O_7_/graphene/CNTs hybrid nanocomposites as anode materials for Li-ion batteries. Diamond Relat. Mater..

[cit22] Yonchai C., Spencer-Jolly D., Palasak P., Pakawanit P., Phatthanakun R., Kidkhunthod P. (2026). *et al.*, Cellulose Nanofiber-MWCNT Buckypaper Engineered Composite Current Collector/Electrode for Flexible and Long-Lasting Semi-Solid-State Li Batteries. J. Alloys Compd..

[cit23] Protyai M. I. H., Saif S. J. A., Rahman M. M., Mural H., Rashid A. B. (2026). An Overview of Recent Progress of Green Nano-Composites for Sustainable Energy Storage Applications. Eng. Rep..

[cit24] Zhang F., Deng N., Wang H., Lu Y., Zhang J., Liu C. (2026). *et al.*, Reaction Mechanisms, Design Principles, and Engineering Strategies of Metal Halides in High-Performance Lithium Rechargeable Batteries. Adv. Funct. Mater..

[cit25] Reddy R. C. K., Lin J., Chen Y. Y., Zeng C. H., Lin X. M., Cai Y. P., Su C. Y. (2020). Progress of nanostructured metal oxides derived from metal-organic frameworks as anode materials for lithium-ion batteries. Coord. Chem. Rev..

[cit26] Baughman R. H. (2002). *et al.*, Carbon nanotubes--the route toward applications. Science.

[cit27] Frackowiak E., Béguin F. (2001). Carbon materials for the electrochemical storage of energy in capacitors. Carbon.

[cit28] Li Q. M., Han N., Chai J. L., Zhang W., Du J. K., Tian H., Liu H., Wang G. X., Tang B. H. J. (2023). Strategies to improve metal-organic frameworks and their derived oxides as lithium storage anode materials. Energy.

[cit29] Eatemadi A. (2014). *et al.*, Carbon nanotubes: properties, synthesis, purification, and medical applications. Nanoscale Res. Lett..

[cit30] Dai L. (2012). *et al.*, Functionalization of graphene: covalent and non-covalent approaches, derivatives and applications. Chem. Rev..

[cit31] Shen M. H., Ma H. L. (2022). Metal-organic frameworks (MOFs) and their derivative as electrode materials for lithium-ion batteries. Coord. Chem. Rev..

[cit32] Li J. (2018). *et al.*, Metal–Organic Framework Templated Pd@PdO–Co_3_O_4_ Nanocubes as an Efficient Bifunctional Oxygen Electrocatalyst. Adv. Energy Mater..

[cit33] Ye C. W., Xu L. (2021). Heteroatom-doped porous carbon derived from zeolite imidazole framework/polymer core-shell fibers as an electrode material for supercapacitor. Composites, Part B.

[cit34] Lee S. S., Kang D. W., Kwak J. H., Shin S., Park J. W., Yu S. H., Jung H. G., Kim B. G., Lim H. D. (2022). Gold-incorporated porous hollow carbon nanofiber for reversible magnesium-metal batteries. Chem. Eng. J..

[cit35] Sun R. L., Yan G. L., Zhang X. L., Li Z. Y., Chen J. Y., Wang L., Wu Y. P., Wang Y. Q., Li H. (2023). Fe-ZIF-derived hollow porous carbon nanofibers for electromagnetic wave absorption. Chem. Eng. J..

[cit36] Nzereogu P. U., Omah A. D., Ezema F. I., Iwuoha E. I., Nwanya A. C. (2022). Anode materials for lithium-ion batteries: a review. Appl. Surf. Sci. Adv..

[cit37] Roy P., Srivastava S. K. (2015). Nanostructured anode materials for lithium-ion batteries. J. Mater. Chem. A.

[cit38] Osiak M., Geaney H., Armstrong E., O'Dwyer C. (2014). Structuring materials for lithium-ion batteries: advancements in nanomaterial structure, composition, and defined assembly on cell performance. J. Mater. Chem. A.

[cit39] Liu Q., Ji Y. X., Yin X. M., Li J. W., Liu Y. J., Hu X., Wen Z. H. (2022). Magnesiothermic reduction improved route to high-yield synthesis of interconnected porous Si@C networks anode of lithium ions batteries. Energy Storage Mater..

[cit40] Li B., Zhang T., Wei S. H., Gao W. (2022). Nitrogen-doped carbon hollow spheres packed with multiple nano Sn particles for enhanced lithium storage. Chem. Eng. J..

[cit41] Su A. Y., Li J., Dong J. J., Yang D., Chen G., Wei Y. J. (2020). An amorphous/crystalline incorporated Si/SiO_*x*_ anode material derived from biomass corn leaves for lithium-ion batteries. Small.

[cit42] Ji H. S., Li J., Li S., Cui Y. X., Liu Z. J., Huang M. G., Xu C., Li G. C., Zhao Y., Li H. M. (2022). High value utilization of silicon cutting waste and excrementum bombycis to synthesize silicon-carbon composites as anode materials for Li-ion batteries. Nanomaterials.

[cit43] Weng C. C., Huang S. M., Lu T., Li J. F., Li J. L., Li J. B., Pan L. K. (2023). NiM (Sb, Sn)/N-doped hollow carbon tube as high-rate and high-capacity anode for lithium-ion batteries. J. Colloid Interface Sci..

[cit44] Xie W., Yun X. R., Lu D., Song W. J., Gao H. J., Guo Q. P., Han Y., Wang H., Xiao P. T., Chen Y. F., Zheng C. M. (2023). Structural engineering of SnSe encapsulated in nitrogen doped carbon nanotubes for high-performance lithium-ion battery anodes. ACS Sustain. Chem. Eng..

[cit45] Ye Y., Wang H. J., Liu H. X., Xiang Y. E., Liu L., Deng W. T., Zou G. Q., Liu Y. C., Hou H. S., Ji X. B. (2022). Carbon dots-regulated pomegranate-like metal oxide composites: from growth mechanism to lithium storage. Small Methods.

[cit46] Pan Z. Y., Sun H., Pan J., Zhang J., Wang B. J., Peng H. S. (2018). The creation of hollow walls in carbon nanotubes for high-performance lithium-ion batteries. Carbon.

[cit47] Fu H. Y., Gao B., Qiao Y., Zhu W. H., Liu Z., Wei G. Y., Feng Z. B., Kamali A. R. (2023). Graphene nanonetwork embedded with polyaniline nanoparticles as anode of Li-ion battery. Chem. Eng. J..

[cit48] Fu S. T., Yu X. F., Wu Q. L., Yang X. F., Liu Z., Li X. H., He S. M., Wang D., Li Y. C., Tong S. F., Wu M. M. (2021). Ultrathin 110-confined Li_4_Ti_5_O_12_ nanoflakes for high rate lithium storage. Adv. Energy Mater..

[cit49] Guo C. Y., Liu Z., Han K., Zhang L. Y., Ding X. L., Wang X. P., Mai L. (2022). Nano-sized niobium tungsten oxide anode for advanced fast-charge lithium-ion batteries. Small.

[cit50] Yuan X. L., Ma Z. T., Jian S. F., Ma H., Lai Y. A., Deng S. L., Tian X. C., Wong C. P., Xia F., Dong Y. F. (2022). Mesoporous nitrogen-doped carbon MnO_2_ multichannel nanotubes with high performance for Li-ion batteries. Nano Energy.

[cit51] Liang Z. Y., Tu H. Y., Kong Z., Yao X. G., Xu D. Q., Liu S. F., Shao Y. L., Wu Y. Z., Hao X. P. (2022). Urchin-like inverse spinel manganese-doped NiCo_2_O_4_ microspheres as high-performance anode for lithium-ion batteries. J. Colloid Interface Sci..

[cit52] Zhao Y. T., Riaz M. S., Dong W. J., Yin Y. F., Liu Z. C., Huang F. Q. (2020). Cu-dispersed cobalt oxides as high volumetric capacity anode materials for Li-ion storage. Energy Storage Mater..

[cit53] Zhang Y. F., Xie M. H., He Y. B., Zhang Y. M., Liu L. D., Hao T. Q., Ma Y., Shi Y. F., Sun Z. J., Liu N., Zhang J. (2021). Hybrid NiO/Co_3_O_4_ nanoflowers as high-performance anode materials for lithium-ion batteries. Chem. Eng. J..

[cit54] Wang F., Li T. F., Fang Y., Wang Z. J., Zhu J. F. (2021). Heterogeneous structured Mn_2_O_3_/Fe_2_O_3_ composite as anode material for high performance lithium-ion batteries. J. Alloys Compd..

[cit55] Xiao B., Wu G., Wang T. D., Wei Z. G., Sui Y. W., Shen B. L., Qi J. Q., Wei F. X., Zheng J. C. (2022). High-entropy oxides as advanced anode materials for long-life lithium-ion batteries. Nano Energy.

[cit56] Liu X. L., Tao R. M., Li C., Wang J. X., Yao S. H., Hong C., Li H. Y., Geng J. Z., Liang J. Y. (2024). Inert salt-assisted solvent-free synthesis of high-entropy oxide towards high performance lithium-ion batteries. Chem. Eng. J..

[cit57] Khan N., Han G., Mazari S. A. (2022). Carbon nanotubes-based anode materials for potassium ion batteries: a review. J. Electroanal. Chem..

[cit58] Kaewraung W., Hasin P. (2024). Germanium-iron alloy particle/multiwalled carbon nanotube composite anode by milling-assisted covalent-bonding for high-performance and stable-cycling lithium-ion batteries. J. Energy Storage.

[cit59] Nandihalli N. (2024). A Review of Nanocarbon-Based Anode Materials for Lithium-Ion Batteries. Crystals.

[cit60] Kiran L. (2023). *et al.*, Mesoporous Cr_2_O_3_/MWCNTs/PANI nanocomposite as a high-performance anode material for rechargeable lithium-ion batteries. Fuel.

[cit61] Zhao Y. L., Stoddart J. F. (2009). Noncovalent functionalization of single-walled carbon nanotubes. Acc. Chem. Res..

[cit62] Dai H. (2002). Carbon nanotubes: opportunities and challenges. Surf. Sci..

[cit63] Shin H.-C., Liu M., Sadanadan B., Rao A. M. (2002). Electrochemical insertion of lithium into multi-walled carbon nanotubes prepared by catalytic decomposition. J. Power Sources.

[cit64] Zhang D., Zhao Y., Goodenough J. B., Lu Y., Chen C., Wang L., Liu J. (2011). Exfoliation from carbon nanotubes *versus* tube size on lithium insertion. Electrochem. Commun..

[cit65] Kang C., Lahiri I., Baskaran R., Kim W.-G., Sun Y.-K., Choi W. (2012). 3-dimensional carbon nanotube for Li-ion battery anode. J. Power Sources.

[cit66] Welna D. T., Qu L., Taylor B. E., Dai L., Durstock M. F. (2011). Vertically aligned carbon nanotube electrodes for lithium-ion batteries. J. Power Sources.

[cit67] Wang H., Nie G. D., Wang Z. Y., Cui S. T., Li H. W., Dang L. Y., Pu Z. P., Liu X. H., Fu A. P., Guo Y. G., Li H. L. (2023). Carbon nanofibers with uniformly embedded silicon nanoparticles as self-standing anode for high-performance lithium-ion battery. Colloids Surf., A.

[cit68] Tong Z., Lv C., Bai G. D., Yin Z. W., Zhou Y., Li J. T. (2025). A review on applications and challenges of carbon nanotubes in lithium-ion battery. Carbon Energy.

[cit69] Meng X., Huang J., Zhu G., Xu Y., Zhu S., Li Q., Chen M., Lin M. C. (2022). Fe_2_O_3_ nanoparticles anchored on thermally oxidized MWCNTs as anode material for lithium-ion battery. Nanotechnology.

[cit70] Li X., Gu H., Liu J., Wei H., Qiu S., Fu Y., Lv H., Lu G., Wang Y., Guo Z. (2015). Multi-walled carbon nanotubes composited with nanomagnetite for anodes in lithium ion batteries. RSC Adv..

[cit71] Ji H., Han Z., Li X., Xu X., Yu S., Yuan L., Li M., Ji R., Tang K. (2025). Synthesis of MWCNT-Si@Ag@CN composite with improved lithium storage performance from photovoltaic kerf loss Si waste. Ionics.

[cit72] Wang H., Nie G. D., Wang Z. Y., Cui S. T., Li H. W., Dang L. Y., Pu Z. P., Liu X. H., Fu A. P., Guo Y. G., Li H. L. (2023). Carbon nanofibers with uniformly embedded silicon nanoparticles as self-standing anode for high-performance lithium-ion battery. Colloids Surf., A.

[cit73] Huang S., Fedorov R. G., Ein-Eli Y. (2024). Silicon-coated multi-walled carbon nanotube (MWCNT) tissues as flexible free-standing anodes for advanced Li-ion batteries. J. Solid State Electrochem..

[cit74] He J., Yang L., Ruan X., Liu Z., Liao K., Duan Q., Zhan Y. (2024). Electrospun PVDF-based polymers for lithium-ion battery separators: a review. Polymers.

[cit75] Im J., Jeong Y. H., Kim M. C., Oh D., Son J., Hyun K., Jeong B., Hong S., Lee J. (2024). Wet spinning of multi-walled carbon nanotube fibers. Carbon.

[cit76] Kim J. S., Baek I. G., Nyamaa O., Goo K. M., Uyanga N., Kim K. S., Noh J. P. (2025). Flexible, binder-free, freestanding silicon/oxidized carbon nanotubes composite anode for lithium-ion batteries with enhanced electrochemical performance through chemical reduction. Mater. Sci. Eng., B.

[cit77] Liu B., Sun X., Liao Z., Lu X., Zhang L., Hao G. P. (2021). Nitrogen and boron doped carbon layer coated multiwall carbon nanotubes as high performance anode materials for lithium ion batteries. Sci. Rep..

[cit78] Zhou Z., Li Z., Liu X., Wu J., Li K., Wang C. (2025). Enhanced performance of yolk-shell SiO/MWCNTs@C composites bridged by MWCNTs for high-performance lithium-ion battery anodes. Chem. Eng. J..

[cit79] Qi W., Shapter J. G., Wu Q., Yin T., Gao G., Cui D. (2017). Nanostructured anode materials for lithium-ion batteries: principle, recent progress and future perspectives. J. Mater. Chem. A.

[cit80] Wang R., Xu C., Sun J., Gao L., Lin C. (2013). Flexible free-standing hollow Fe_3_O_4_/graphene hybrid films for lithium-ion batteries. J. Mater. Chem. A.

[cit81] Zhang G., Li J., Sha J., He C., Liu E., Zhao N., Shi C. (2016). Preparation of Fe_3_O_4_/rebar graphene composite *via* solvothermal route as binder free anode for lithium-ion batteries. J. Alloys Compd..

[cit82] Zou M., Li J., Wen W., Chen L., Guan L., Lai H., Huang Z. (2014). Direct formic acid microfluidic fuel cell design and performance evolution. J. Power Sources.

[cit83] Li X. H., Xu K., Yu Y. J., Chen C., Zhang H. J., Lei W. (2024). Self-standing high performance Si nanoparticle anchored porous carbon fiber composites for Li-ion batteries. Electrochim. Acta.

[cit84] Yin H., Li H. Y., Zhao H., Gan Z. S., Hou Z. H., Li C., Zhu M. Q. (2023). SnSe nanoplates swallowed in carbon nanofibers as the self-standing anode for lithium-ion and sodium batteries with enhanced performance. Int. J. Hydrogen Energy.

[cit85] Nandihalli N. (2024). A Review of Nanocarbon-Based Anode Materials for Lithium-Ion Batteries. Crystals.

[cit86] Chen Y. (2021). *et al.*, A Comprehensive Review on Metal-Oxide Nanocomposites for High-Performance Lithium-Ion Battery Anodes. Energy Fuels.

[cit87] Kang Y.-M., Song M.-S., Kim J.-H., Kim H.-S., Park M.-S., Lee J.-Y., Liu H. K., Dou S. X. (2005). Effect of Cl^−^ ions and benzylideneacetone/ethanol on the underpotential deposition of zinc in acidic media: cyclic voltammetry and EQCM studies. Electrochim. Acta.

[cit88] Gao L., Luo S. B., Li J. X., Cheng B. W., Kang W. M., Deng N. P. (2021). Core–shell structure nanofibers-ceramic nanowires based composite electrolytes with high Li transference number for high-performance all-solid-state lithium metal batteries. Energy Storage Mater..

[cit89] Bharti V. K., Goswami A. P., Sharma C. S., Khandelwal M. (2024). Bacterial cellulose derived carbon as a self-supported and flexible anode for stable-performance lithium-ion batteries. J. Electroanal. Chem..

[cit90] Sun Z., Madej E., Wiktor C., Sinev I., Fischer R. A., van Tendeloo G., Muhler M., Schuhmann W. (2015). Release of the Water Molecule Encapsulated Inside an Open-Cage Fullerene through Hydrogen Bonding Mediated by Hydrogen Fluoride. Chem.–Eur. J..

[cit91] Gao G., Zhang Q., Wang K., Song H., Qiu P., Cui D. (2013). Reactivation of dissolved polysulfides in Li–S batteries based on atomic layer deposition of Al_2_O_3_ in nanoporous carbon cloth. Nano Energy.

[cit92] Zhou X. H., Su K. M., Kang W. M., Cheng B. W., Li Z. H., Jiang Z. (2020). Locking metal sulfide nanoparticles in interconnected porous carbon nanofibers with protective macro-porous skin as freestanding anodes for lithium ion batteries. Chem. Eng. J..

[cit93] Peng J. J., Tao J., Liu Z. J., Yang Y. H., Yu L., Zhang M., Wang F., Ding Y. (2021). Ultrastable and high capacity flexible lithium-ion batteries based on bimetallic MOFs derivatives aiming for wearable electronic devices. Chem. Eng. J..

[cit94] Pang X. J., Zhang J., Qi G. W., Dai X. H., Zhou J. P., Zhang S. Y. (2015). Multi-walled carbon nanotube-reinforced porous iron oxide as a superior anode material for lithium ion battery. J. Alloys Compd..

[cit95] Zhang Z. H., Ying H. J., Huang P. F., Zhang S. L., Zhang Z., Yang T. T., Han W. Q. (2023). Porous Si decorated on MXene as free-standing anodes for lithium-ion batteries with enhanced diffusion properties and mechanical stability. Chem. Eng. J..

[cit96] Zhang T., Qiu D. P., Hou Y. L. (2022). Free-standing and consecutive ZnSe@carbon nanofibers architectures as ultra-long lifespan anode for flexible lithium-ion batteries. Nano Energy.

[cit97] Guler M. O., Cetinkaya T., Uysal M., Akbulut H. (2015). High efficiency TiO_2_/MWCNT based anode electrodes for Li-ion batteries. Int. J. Energy Res..

[cit98] Ma F., Yuan A., Xu J., Hu P. (2015). Porous α-MoO_3_/MWCNT nanocomposite synthesized *via* a surfactant-assisted solvothermal route as a lithium-ion-battery high-capacity anode material with excellent rate capability and cyclability. ACS Appl. Mater. Interfaces.

[cit99] Ghannam M. M., Heiba Z. K., Sanad M. M., Mohamed M. B. (2020). Functional properties of ZnMn_2_O_4_/MWCNT/graphene nanocomposite as anode material for Li-ion batteries. Appl. Phys. A.

[cit100] Chen Y., Du N., Zhang H., Yang D. (2015). Facile synthesis of uniform MWCNT@Si nanocomposites as high-performance anode materials for lithium-ion batteries. J. Alloys Compd..

[cit101] Chen Y., Mao Q., Bao L., Yang T., Lu X., Du N., Ji Z. (2018). Rational design of coaxial MWCNTs@ Si/SiO_*x*_@C nanocomposites as extending-life anode materials for lithium-ion batteries. Ceram. Int..

[cit102] Xu J., Liu Y., He L., Zhang C., Zhang Y. (2016). Facile synthesis of CuO mesocrystal/MWCNT composites as anode materials for high areal capacity lithium ion batteries. Ceram. Int..

[cit103] Liu H., Li X., Xing G. (2020). Preparation and electrochemical performance of MnO_2_/Fe_3_O_4_/MWCNTs nanocomposites as anode for a lithium-ion battery. Int. J. Electrochem. Sci..

[cit104] Tan Y., Zhu K., Li D., Bai F., Wei Y., Zhang P. (2014). Performance and hydrodynamic features of a staged up-flow ANAMMOX sludge bed (SUASB) reactor. Chem. Eng. J..

[cit105] He X. J., Wang X. D., Tang M., Zhang H., Wang Y. (2022). Self-supporting ZnP_2_@N, P codoped carbon nanofibers as high-performance anode material for lithium-ion batteries. J. Alloys Compd..

[cit106] Shu J., Li H., Yang R., Shi Y., Huang X. (2006). Novel electrode structure for DMFC operated with liquid methanol. Electrochem. Commun..

[cit107] Wen K., Wang K., Chen L., Xie J. (2006). Self-assembled monolayers on bismuth electrodes. Electrochem. Commun..

[cit108] Ding Y. Y., Li P. P., Wang J. S., Li X., Liu Y., Bai H. C., Zhang H. (2023). Rationally designed rGO@CNTs@CNFs film as self-supporting binder-free Si electrodes for high performance lithium-ion batteries. J. Colloid Interface Sci..

[cit109] Xie S. M., Yao T. H., Wang J. K., Alsulami H., Kutbi M. A., Wang H. K. (2020). Coaxially integrating TiO_2_/MoO_3_ into carbon nanofibers *via* electrospinning towards enhanced lithium ion storage performance. ChemistrySelect.

[cit110] Chen Z. H., Wang T., Liu Y., Liu Y. C., Yang X. P. (2024). Hydrogen-titanate-protected SnO_2_@C coaxial nanocables as new nanocomposite anode materials for lithium ion batteries. Chem. Eng. J..

[cit111] Singh E., Singh B. P., Bijoy T. K., Reddy V. R., Karthikeyan G., Singh V. N., Dhakate S. R., Murugan P., Gopukumar S. (2017). *In situ* conversion of multiwalled carbon nanotubes to graphene nanosheets: an increasing capacity anode for Li ion batteries. Electrochim. Acta.

[cit112] Köse H., Aydin A. O., Akbulut H. (2013). Sol–gel preparation and electrochemical characterization of SnO_2_/MWCNTs anode materials for Li-ion batteries. Appl. Surf. Sci..

[cit113] Hong S.-H., Kang H., Kim T.-H., Choi S., Oh J.-H. (2024). Thermal plasma synthesis of Si-multi walled carbon nanotube nanocomposite as an anode for lithium-ion batteries. Thin Solid Films.

[cit114] Kim J. S., Baek I. G., Nyamaa O., Goo K. M., Uyanga N., Kim K. S., Nam T. H., Yang J. H., Noh J. P. (2025). Flexible, binder-free, freestanding silicon/oxidized carbon nanotubes composite anome for lithium-ion batteries with enhanced electrochemical performance through chemical reduction. Mater. Sci. Eng., B.

[cit115] Wang H., Nie G. D., Wang Z. Y., Cui S. T., Li H. W., Dang L. Y., Pu Z. P., Liu X. H., Fu A. P., Guo Y. G., Li H. L. (2023). Carbon nanofibers with uniformly embedded silicon nanoparticles as self-standing anode for high-performance lithium-ion battery. Colloids Surf., A.

[cit116] Jiang R. Q., Yuan H. C., Wei X. B., Wang H. J., Shin H. J., Lan J. L., Yu Y. H., Yang X. P. (2021). Constructing robust and freestanding MXene/Si@C core-shell nanofibers *via* coaxial electrospinning for high performance Li-ion batteries. Mater. Chem. Front..

[cit117] Hong S.-H., Kim T.-H., Oh J.-H., Choi S. (2024). Thermal plasma synthesis of Si-MWCNT nanocomposite as an anode for lithium-ion batteries. Thin Solid Films.

[cit118] Sun L., Liu Y., Shao R., Wu J., Jiang R. Y., Jin Z. (2022). Recent progress and future perspective on practical silicon anode-based lithium ion batteries. Energy Storage Mater..

[cit119] Li P., Zhao G., Zheng X., Xu X., Yao C., Sun W., Dou S. X. (2018). Recent progress on silicon-based anode materials for practical lithium-ion battery applications. Energy Storage Mater..

[cit120] Pei Y., Wang Y., Chang A. Y., Liao Y., Zhang S., Wen X., Wang S. (2023). Nanofiber-in-microfiber carbon/silicon composite anode with high silicon content for lithium-ion batteries. Carbon.

[cit121] Ng S. H., Wang J., Wexler D., Chew S. Y., Liu H. K. (2007). Amorphous carbon-coated silicon nanocomposites: a low-temperature synthesis *via* spray pyrolysis and their application as high-capacity anodes for lithium-ion batteries. J. Phys. Chem. C.

[cit122] Zhang H. R., Qin X. Y., Wu J. X., He Y. B., Du H. D., Li B. H., Kang F. Y. (2015). Electrospun core–shell silicon/carbon fibers with an internal honeycomb-like conductive carbon framework as an anode for lithium ion batteries. J. Mater. Chem. A.

[cit123] Li C. H., Yuan C. S., Zhu J. Y., Ni X. P., Li K. M., Wang L., Qi Y. J., Ju A. Q. (2022). Fabrication of silicon nanoparticles/porous carbon@porous carbon nanofibers core-shell structured composites as high-performance anodes for lithium-ion batteries. Colloids Surf., A.

[cit124] Akbulut H., Nalci D., Guler A., Duman S., Guler M. O. (2018). Carbon–silicon composite anode electrodes modified with MWCNT for high energy battery applications. Appl. Surf. Sci..

[cit125] Zeng Q., Tian S. H., Liu G., Yang H. C., Sun X., Wang D., Huang J. J., Yan D., Peng S. L. (2022). Sulfur-bridged bonds boost the conversion reaction of the flexible self supporting MnS@MXene@CNF anode for high-rate and long-life lithium-ion batteries. ACS Appl. Mater. Interfaces.

[cit126] Hou T., Liu B., Sun X., Fan A., Xu Z., Cai S., Zheng C., Yu G., Tricoli A. (2021). Covalent coupling-stabilized transition-metal sulfide/carbon nanotube composites for lithium/sodium-ion batteries. ACS Nano.

[cit127] Muralidharan N., Nallathamby K. (2021). CuCo_2_S_4_: versatile anode for high capacity and high rate for lithium and sodium ion battery application. J. Phys. Chem. Solids.

[cit128] Zhang W. X., Li Z., Zhang J. H., He C., Ning X. H. (2024). NiS_2_@MWCNTs as a promising anode material for lithium and sodium-ion batteries with superior cycling stability. J. Alloys Compd..

[cit129] Yoo H., Tiwari A. P., Lee J., Kim D., Park J. H., Lee H. (2015). Cylindrical nanostructured MoS_2_ directly grown on CNT composites for lithium-ion batteries. Nanoscale.

[cit130] Hu D., Zhou Y., Wu J., Zhang Y., Weng Z., Chang X., Chen W. (2024). Interfacial chemical linkage enabling MWCNTs/Sb_2_S_3_ high sodium storage performance. Energy Fuels.

[cit131] Zhou Y., Yan D., Xu H., Liu S., Yang J., Qian Y. (2015). Multiwalled carbon nanotube@aC@Co_9_S_8_ nanocomposites: a high-capacity and long-life anode material for advanced lithium ion batteries. Nanoscale.

[cit132] Li X. Q., Guan G. G., Cheng B. J., Zhang X. K., Zhang K. Y., Xiang J. (2024). Well-dispersed FeNi nanoparticles embedded in N-doped
carbon nanofiber membrane as a self supporting and binder-free anode for lithium-ion batteries. J. Mater. Chem. C.

[cit133] Lahiri I., Oh S. W., Hwang J. Y., Cho S., Sun Y. K., Banerjee R., Choi W. (2010). High capacity and excellent stability of lithium ion battery anode using interface-controlled binder-free multiwall carbon nanotubes grown on copper. ACS Nano.

[cit134] Di Lecce D., Andreotti P., Boni M., Gasparro G., Rizzati G., Hwang J. Y., Sun Y. K., Hassoun J. (2018). Multiwalled carbon nanotubes anode in lithium-ion battery with LiCoO_2_, Li[Ni_1/3_Co_1/3_Mn_1/3_]O_2_, and LiFe_1/4_Mn_1/2_Co_1/4_PO_4_ cathodes. ACS Sustain. Chem. Eng..

[cit135] Ahmed F., Kumar S., Shaalan N. M., Saber O., Rehman S., Aljaafari A., Abuhimd H., Alshahrani M. (2022). Synergistic effect of hexagonal boron nitride-coated separators and multi-walled carbon nanotube anodes for thermally stable lithium-ion batteries. Crystals.

[cit136] Srivastava G., Dalela S., Gautam N. K., Kumar S., Hashmi S. Z., Ahmad M. A., Quraishi A. M., Khanna V., Alvi P. A. (2025). Synthesis and characterizations of MnO_2_/CNT nanocomposite for usage as electrodes in high-performance supercapacitor. Nano Trends.

